# Manganese based layered oxides with modulated electronic and thermodynamic properties for sodium ion batteries

**DOI:** 10.1038/s41467-018-07646-4

**Published:** 2019-01-07

**Authors:** Kai Zhang, Duho Kim, Zhe Hu, Mihui Park, Gahee Noh, Yujeong Yang, Jing Zhang, Vincent Wing-hei Lau, Shu-Lei Chou, Maenghyo Cho, Si-Young Choi, Yong-Mook Kang

**Affiliations:** 1 Department of Energy and Materials Engineering, Dongguk University-Seoul, Seoul, 04620 South Korea; 2 Department of Mechanical and Aerospace Engineering, Seoul National University, Gwanak-ro 1, Gwanak-gu, Seoul 08826 South Korea; 3 Institute for Superconducting and Electronic Materials, University of Wollongong, Wollongong, New South Wales 2522 Australia; 4 Department of Materials Science & Engineering, POSTECH, 77 Cheongam-ro, Nam-gu, Pohang 37673 South Korea; 5 Department of Mechanical Engineering, Kyung Hee University, 1732, Deogyeong-daero, Giheung-gu, Yongin-si, Gyeonggi-do, 17104 Republic of Korea

## Abstract

Manganese based layered oxides have received increasing attention as cathode materials for sodium ion batteries due to their high theoretical capacities and good sodium ion conductivities. However, the Jahn–Teller distortion arising from the manganese (III) centers destabilizes the host structure and deteriorates the cycling life. Herein, we report that zinc-doped Na_0.833_[Li_0.25_Mn_0.75_]O_2_ can not only suppress the Jahn–Teller effect but also reduce the inherent phase separations. The reduction of manganese (III) amount in the zinc-doped sample, as predicted by first-principles calculations, has been confirmed by its high binding energies and the reduced octahedral structural variations. In the viewpoint of thermodynamics, the zinc-doped sample has lower formation energy, more stable ground states, and fewer spinodal decomposition regions than those of the undoped sample, all of which make it charge or discharge without any phase transition. Hence, the zinc-doped sample shows superior cycling performance, demonstrating that zinc doping is an effective strategy for developing high-performance layered cathode materials.

## Abstract (web summary)

Mn-based layered oxides are promising cathode materials for next generation sodium ion batteries. To address two existing issues facing the system, here the authors show that a simple zinc doping can suppress both Jahn–Teller distortion and phase separation, enabling enhanced cycling performance.

## Introduction

The gradual depletion of fossil fuels, together with environmental concerns over their use, has led to a rapidly increasing demand for high performance electrochemical energy storage and conversion (EESC) technologies, driving the development of secondary batteries^1–5^. Among various EESC devices, lithium-ion batteries (LIBs) have the widest applications in various fields^6–9^. However, limited lithium availability makes it difficult to meet the forecast growth in market demand for LIBs. In contrast, Na is widely distributed around the world and is inexpensive, making sodium-ion batteries (SIBs) a promising alternative for LIBs^10–18^.

As cathode materials are the key determinant for the performance of SIBs, various compounds have been investigated as SIB cathodes, including sodium transition-metal oxides, polyanions, and alkali-metal hexacyanometalates^19–22^. Among these materials, sodium-based layered transition-metal oxides (abbreviated as Na_*x*_MO_2_ where M is a first row transition-metal) have gained significant attention because of their unique structural advantages and high theoretical capacities^23^. To date, O3-type and P2-type layered structures have been intensely studied among various sodium-based layered transition-metal oxides, although there is greater interest in the latter. This is because P2-type Na_*x*_MO_2_ generally performs better electrochemically due to its lower Na^+^ diffusion barrier and higher ionic conductivity than its O3 phase^23,24^. In addition, O3-phase samples are too hygroscopic to be stored at ambient atmosphere^25^, while P2-phase has sufficient stability to be stored at ambient condition.

Mn-based layered oxides have been extensively studied for SIBs due to their low costs, elemental availability and environmental friendliness^13,26,27^. However, these materials tend to undergo large Jahn–Teller distortions induced by Mn^3+^ ions (*t*_*2g*_^3^*e*_*g*_^1^), leading to severe cyclic degradations during charge or discharge. Unlike Mn^3+^ ions, Mn^4+^ ions (*t*_*2g*_^3^*e*_*g*_^0^) occupy the octahedral sites and stabilize their electronic structures based on energetics^28,29^. In order to suppress the Jahn–Teller distortion for cyclic improvement, several research groups focused on doping different metal ions (e.g., Mg, Al, and Li) into Mn-based layered oxides^30–34^. Even though such metal doping helped to enhance capacity retention, their capacity retentions are still insufficient for the practical application of Mn-based layered oxides as conventional cathode materials. Furthermore, due to the larger ionic radius of Na^+^ ion, Na_*x*_MO_2_ generally undergoes more drastic phase transformations during desodiation than LiMO_2_ during delithiation, which makes its degradation more disastrous^35–37^.

Hence, reducing the Jahn–Teller distortion and controlling the phase transformations in Na_*x*_MO_2_ during repeated charge/discharge look critical for its use as a conventional cathode material. Some studies attempted to understand their electronic structure and thermodynamic phase stability upon Na extraction using both theoretical and experimental approaches, because this stability is directly associated with the capacity retention of Na_*x*_MO_2_^38–40^. In this regard, reducing the amount of Mn^3+^ and suppressing the phase separation during desodiation may be essential for improving the capacity retention of Mn-based layered oxides.

Considering that Jahn–Teller distortion is primarily induced by trivalent Mn ions and the inherent phase transformations of Mn-based layered oxides are closely linked to their thermodynamic instability, divalent Zn has been doped into P2-type Na_0.833_[Li_0.25_Mn_0.75_]O_2_ (NLMO) to investigate its structural and thermodynamic contributions in this study. The reason to choose P2-type NLMO is because introduction of Li into the transition-metal layer can increase the Na content and thus enhance its capacity^32,41^. Specifically, (Na_0.833_Zn_0.0375_)[Li_0.25_Mn_0.7125_]O_2_ (NLMO-Zn) has been obtained via Zn doping by a sol–gel process combined with a subsequent heat treatment. Such doping not only alleviates Jahn–Teller distortion by increasing the average oxidation state of Mn but also reduces the inherent phase separation or instability. This approach has been rationalized using first-principles calculation and has been verified using several analytical techniques, such as X-ray photoelectron spectroscopy (XPS), X-ray absorption spectroscopy (XAS), and in situ X-ray diffraction (XRD). In comparison with bare NLMO, Zn-doped samples have superior capacity retention. These findings can be used to formulate rational strategies to improve the phase stabilities and electrochemical performances of manganese-based cathode materials in both LIBs and SIBs.

## Results

### Theoretical prediction of electronic and thermodynamic stability

**Fig. 1:**
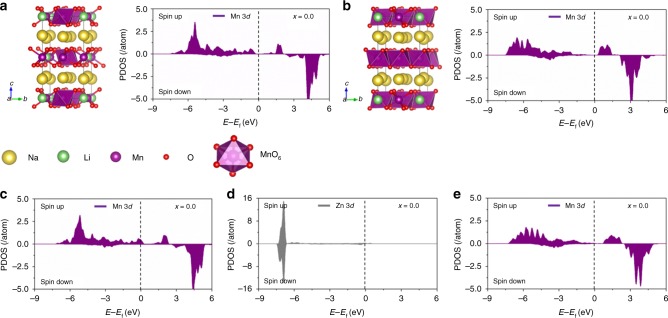
Theoretical calculations regarding the effects of Zn doping in the fully sodiated Na[Li_0.25_Mn_0.75_]O_2_. **a** Shows the Mn^3+^ sites as purple octahedra in the relaxed crystal structure of Na[Li_0.25_Mn_0.75_]O_2_ together with its corresponding PDOS. Likewise, the Mn^4+^ sites are shown as purple octahedra in **b** together with its corresponding PDOS. **c** Shows PDOS of the Mn^3+^ sites in the Zn-doped Na[Li_0.25_Mn_0.75_]O_2_. Likewise, PDOSs of the Zn^2+^ and the Mn^4+^ sites in the Zn-doped Na[Li_0.25_Mn_0.75_]O_2_ are shown in **d**, **e**, respectively

The material we targeted for the Zn doping strategy, Na[Li_0.25_Mn_0.75_]O_2_, is expected to have a mixed Mn valence of 3+ and 4+ to meet the charge balance. We first verified this mixed valence by computationally modelling the structure of Na[Li_0.25_Mn_0.75_]O_2_ and calculating the partial density of states (PDOS) of Mn. As expected, Mn in these oxidation states is present with the Mn^3+^ occupying the sites identified by the purple octahedra (Fig. 1a) and Mn^4+^ by the purple octahedra (Fig. 1b).

We then modelled the Zn-doped Na[Li_0.25_Mn_0.75_]O_2_ structure, and the structure was fully relaxed using first-principles method (Fig. 1c−e). The PDOSs of the Mn^3+^, Zn^2+^, and Mn^4+^ centers are illustrated in Fig. 1c–e, respectively. It is found that the Mn^3+^ centers are oxidized (Fig. 1c), leading to the increased density of states of the Mn^4+^ centers in the Zn-doped model compared to the undoped model. This indicates that Zn-doped Na[Li_0.25_Mn_0.75_]O_2_, which includes more Mn^4+^, would experience less Jahn–Teller distortion and hence better capacity retention compared to the undoped Na[Li_0.25_Mn_0.75_]O_2_.

**Fig. 2:**
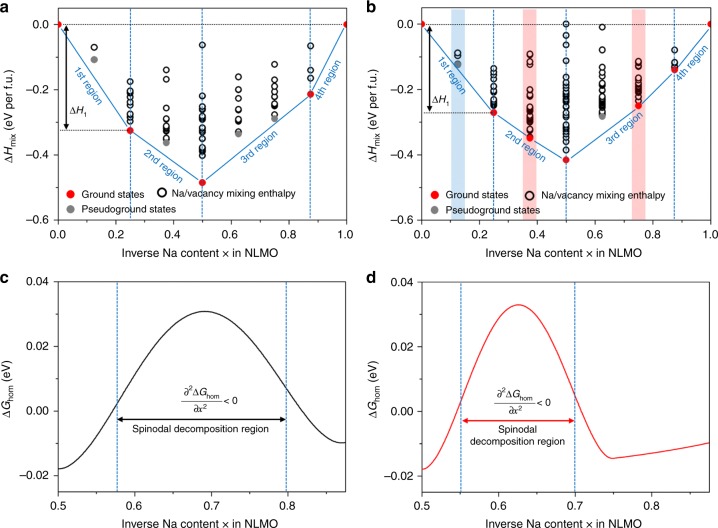
The phase stability of Na[Li_0.25_Mn_0.75_]O_2_ and Zn-doped Na[Li_0.25_Mn_0.75_]O_2_ with different Na contents. The mixing enthalpy (∆*H*_mix_) values were calculated considering all possible Na and their vacancy configurations and the results are shown for Na[Li_0.25_Mn_0.75_]O_2_ (**a**) and Zn-doped Na[Li_0.25_Mn_0.75_]O_2_ (**b**). Here, the two atomic models contain 8 Na atoms in each unit cell structure. Based on statistical thermodynamics, many unit cell structures can be generated based on the combinations of Na and its vacancy sites in the unit cell structure. Among them, one should have the lowest mixing enthalpy value as marked with the red or grey circles in **a** and **b**, while the other structures must have higher enthalpy values (marked with white circles). Above the tie lines (blue solid lines), grey filled circles are notated as pseudo ground states that are not preferential to exist in certain compositions. In the cases where the ground state as indicated by the red circles lie on the tie lines, these are true ground states as their structures are thermodynamically favourable at the specified compositions. ∆*H*_*1*_ indicates the difference between the lowest energy values at *x* = 0.25 and *x* = 0.0. Homogeneous bulk free energies are shown for Na[Li_0.25_Mn_0.75_]O_2_ (**c**) and Zn-doped Na[Li_0.25_Mn_0.75_]O_2_ (**d**) at 300 K. In both panels, the range between the dotted lines indicate the spinodal decomposition regions satisfying the condition of $\frac{{\partial \Delta G_{\hom }}}{{\partial x^2}} < 0$

Because the capacity retention of cathode material is closely associated with its thermodynamic phase stability which is mainly changed during the extraction of charge carriers, the formation energies of mixing enthalpy (∆*H*_mix_) for Na[Li_0.25_Mn_0.75_]O_2_ and Zn-doped Na[Li_0.25_Mn_0.75_]O_2_ were calculated with Na content and shown in Fig. 2a, b. The calculated thermodynamic energy values have been utilized for plenty of battery materials to predict their phase stability as a function of charge carrier content. In these calculations, we have considered all possible combinations of Na ions and their vacancies in Na_1−*x*_[Li_0.25_Mn_0.75_]O_2_ and Zn-doped Na_1−*x*_[Li_0.25_Mn_0.75_]O_2_ when their sodium contents are in the range of *x* = 0.0−1.0. From these energy diagrams, Na_1−*x*_[Li_0.25_Mn_0.75_]O_2_ exhibits five ground states, while Zn-doped Na_1−*x*_[Li_0.25_Mn_0.75_]O_2_ includes seven ground states. In details, both of Fig. 2a, b are composed of four regions with the inverse Na content. In the first region from *x* = 0 to *x* = 0.25, the Zn-doped Na[Li_0.25_Mn_0.75_]O_2_ has a ∆*H*_mix_ difference (notated as ∆*H*_*1*_ in Fig. 2a, b, i.e., ∆*H*_*1*_ *=* ∆*H*_mix_(*x* = 0.25) *−* ∆*H*_mix_(*x* = 0.0)) of −0.2709 eV, which is lower than that of Na[Li_0.25_Mn_0.75_]O_2_ (−0.3256 eV), implying that a volumetric strain (*ε*_V_ = Δ*V*/*V*_*x*=0_ where Δ*V* is the volumetric change and *V*_*x*=0_ is its unit cell volume at *x* = 0) for the Zn-doped structure (*ε*_V_ = −0.027) is smaller than that for the undoped structure (*ε*_V_ = −0.044) during the initial desodiation. In the second region, the ground states of Zn-doped Na[Li_0.25_Mn_0.75_]O_2_ as marked by the red circles in Fig. 2b lie on the tie line between *x* = 0.25 and *x* = 0.5, indicative of desodiation occurring in the same phase. This is in contrast to its undoped counterpart, where the appearance of a pseudo ground state (grey circle) at *x* = 0.38 suggests the formation of a new phase, that is, desodiation involves a two-phase reaction in Na[Li_0.25_Mn_0.75_]O_2_. In the third region (*x* = 0.50–0.88), the two pseudo ground states are shown as grey circle above the tie line at *x* = 0.63 and at *x* = 0.75 are present for Na[Li_0.25_Mn_0.75_]O_2_ and is indicative of phase separation when desodiated to this extent. By contrast, Zn-doped Na[Li_0.25_Mn_0.75_]O_2_ only has one pseudo ground state as illustrated by the grey circle at *x* = 0.63, indicating that phase separation may occur only within the narrow region of *x* = 0.5 and *x* = 0.75. The additional ground states in Zn-doped Na[Li_0.25_Mn_0.75_]O_2_ can contribute to suppressing the inherent phase separation or transition which can occur in Na[Li_0.25_Mn_0.75_]O_2_^42^.

To investigate the detailed phase separations in the third region of the two samples, minimum ∆*H*_mix_ values were fitted using a double-well function. Then, the homogeneous bulk free energies (∆*G*_hom_), which can predict spinodal decomposition region and the related phase separation behaviour^43^, were calculated at 300 K and shown in Fig. 2c, d. For Na[Li_0.25_Mn_0.75_]O_2_, the range of inverse Na content (*x* value) where spinodal decomposition would occur looks broad, suggesting that the pristine sample continues to suffer from a couple of phase separations when charged above *x* = 0.57. Meanwhile, due to the additional ground states accompanied by Zn doping, the doped sample tends to have a spinodal decomposition just within a much narrower range of inverse Na content and thereby remarkably reduces the degree of phase separation when charged above *x* = 0.54. The calculated thermodynamic phase stability predicts that Zn-doped Na[Li_0.25_Mn_0.75_]O_2_ has lower ∆*H*_*1*_ and more stable intermediate phases, and smaller spinodal decomposition region, finally enhancing its cyclic stability compared to Na[Li_0.25_Mn_0.75_]O_2_.

Following these computational findings, we also investigated the phase stability of NLMO and NLMO-Zn at different temperatures and compositions by calculating their bulk free energies. NLMO-Zn has a lower free energy than NLMO at all temperatures and at all extent of sodium extraction (Supplementary Fig. 1), indicating that the doped sample exhibits higher stability than its undoped counterpart.

### Preparation and structural analysis of materials

Following computational verification of our hypothesis, we proceeded to synthesize undoped NLMO and NLMO-Zn using a sol–gel method combined with a subsequent heat treatment. The precursor solution was evaporated at 80 °C for 12 h and was then heated in a vacuum oven at 60 °C for 4 h, yielding a viscous white mixture (Supplementary Fig. 2). This mixture was pre-calcined first for 2 h at 400 °C, the temperature at which all volatiles have been removed according to thermal gravimetric analysis (TGA) (Supplementary Fig. 3), and then re-ground and pelletized for a final calcination at 700 °C for 6 h in air.

**Fig. 3:**
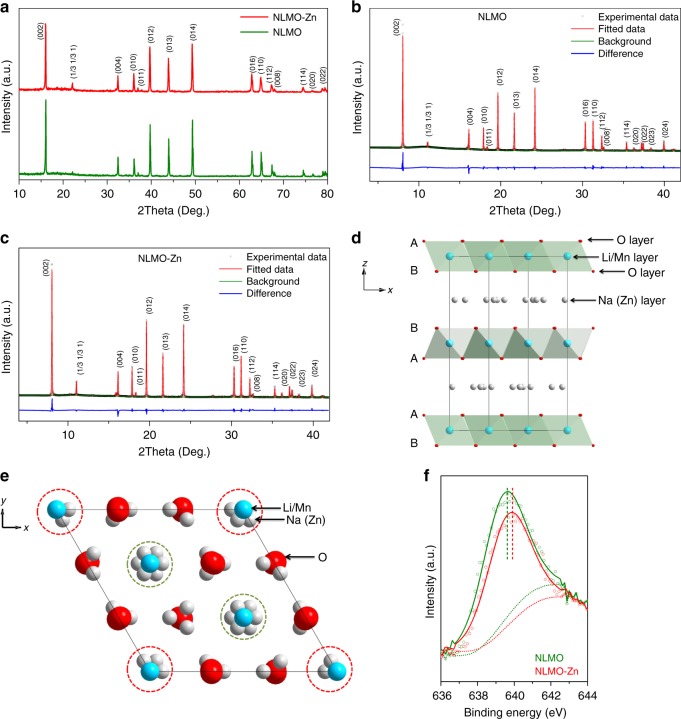
Structural analyses of NLMO and NLMO-Zn. **a** Shows the XRD patterns of NLMO and NLMO-Zn. **b**, **c** Show the Rietveld refinement results for high-resolution diffraction patterns (HRPD) of NLMO and NLMO-Zn, respectively. Crystal structure of P2-type NLMO (or NLMO-Zn) viewed along the *y*-axis (**d**) and the *z*-axis (**e**). **f** Shows the XPS spectra in the Mn 2*p*3/2 region of NLMO and NLMO-Zn

Figure 3a shows the XRD patterns of NLMO and NLMO-Zn. All peaks in both samples agree well with the pattern having the P2-phase from previous studies (Supplementary Fig. 4)^32^, demonstrating that the P2-type structure is constructed without any impurity phase. For more accurate structure determination of the doped and undoped NLMO, high resolution powder diffraction (HRPD) patterns were collected using synchrotron X-ray with a wavelength of 0.7749 Å. Figure 3b, c each show the Rietveld refinement results for pristine NLMO and NLMO-Zn. The patterns of both samples have high peak intensities, which indicate their high crystallinity. The detailed cell parameters for NLMO and NLMO-Zn are listed in Table 1 and Supplementary Tables 1, 2. NLMO and NLMO-Zn have a hexagonal structure in the P6_3_ space group; no other phase is found, confirming the phase purity of both samples. The corresponding P2-type crystal structure of NLMO or NLMO-Zn is shown in Fig. 3d, e. When viewed along the y-axis, the layered structure of P2-NLMO where O-layers are stacked in ABBAAB sequence is clearly visible. Therein, Li and Mn ions occupy the same positions (2a and 2b sites) but with different occupancies. When viewed along the z-axis, some Na ions seem to surround O ions, while other Na ions are observed to surround Li and Mn ions. After doping Zn, Zn ions occupy 6c positions in the Na layer. The detailed description of crystal structure of NLMO or NLMO-Zn is placed in Supplementary Note.

**Table 1:** Crystallographic parameters of NLMO and NLMO-Zn, based on Rietveld refinement results

	*a* = *b* (Å)	*c* (Å)	*V* (Å^3^)	*α* = *β*	*γ*	*R*_wp_	*R*_p_	*χ* ^2^
**NLMO**	4.9722(6)	11.0425(7)	236.43	90°	120°	6.94	4.85	3.132
**NLMO-Zn**	4.9925(2)	11.0404(0)	238.32	90°	120°	6.64	4.46	3.452

The electronic structures of NLMO and NLMO-Zn were characterized by XPS, and their spectra in the Mn 2p_3/2_ region are shown in Fig. 2f. The Mn in NLMO-Zn has a higher binding energy, which suggests that the oxidation state of Mn in this sample is closer to 4+ than that in NLMO. Furthermore, the higher binding energy of Zn in NLMO-Zn indicates that the Mn−O bonds in NLMO-Zn are more robust than those in NLMO, which may contribute to improving the phase stability and thereby the capacity retention. These XPS results experimentally confirm our hypothesis and computational design of employing Zn dopant to reduce the amount of the Jahn–Teller distorted Mn^3+^ centers. For completeness, the Zn 2*p* partial spectrum is provided in Supplementary Fig. 5. Here, the Zn 2*p*_3/2_ and 2*p*_1/2_ peaks are located at 1023.13 and 1046.14 eV, respectively, coinciding with those of ZnCl_2_ and ZnSO_4_ (from the National Institute of Standards and Technology (NIST) database) and demonstrating that the doped Zn in NLMO-Zn has a valence very close to +2.

**Fig. 4:**
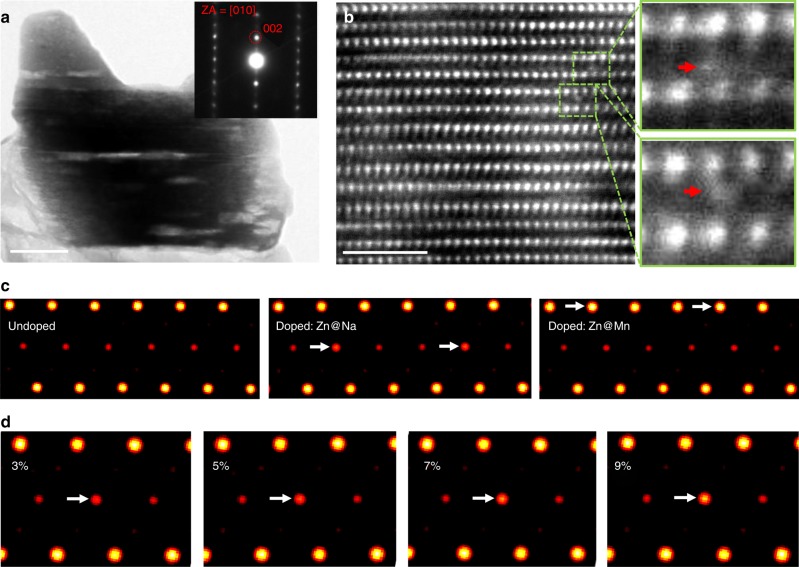
Microscopy structure investigation of NLMO-Zn. **a** Bright-field TEM image of the particle along the [010] projection. Inset shows the selected area diffraction pattern from the particle. **b** HAADF-STEM image of NLMO-Zn. **c** Simulated HAADF images in the [010] projection of the three cases: undoped NLMO, 5%-Zn occupying the Na site in NLMO-Zn, and 5%-Zn occupying the Mn site in NLMO-Zn. **d** Simulated HAADF images showing the different contrasts by the variation of Zn-occupancy (3%, 5%, 7%, and 9% Zn in the Na layer)

In order to directly validate the location of the doped Zn, we conducted scanning transmission electron microscopy (STEM) and solid-state nuclear magnetic resonance (NMR) analyses together. Bright-field TEM image of the NLMO-Zn particle along the [010] projection with the corresponding selected area diffraction pattern (SAED) is shown in Fig. 4a. The P2-type layered structure is verified again, which is consistent with the HRPD refinement results. HAADF-STEM images of NLMO-Zn are displayed in Fig. 4b, and bright contrasts exist in between two adjacent transition-metal layers, which are marked by the red arrows in the magnified images on the right side of panel b. It demonstrates that Zn dopants exist in the Na layers. To further understand the Zn position, a series of HAADF-STEM image simulations were done. As shown in Fig. 4c, Zn located at the Na layer causes a bright contrast, while no significant difference can be observed compared to the undoped case, demonstrating that Zn^2+^ ions are likely to occupy in Na layer. Figure 4d shows that the contrast caused by Zn at the Na layer varied depending on its occupancy. These simulation results indicate that the occupancy of Zn in the Na-atomic columns is less than 10%. The Zn^2+^ ions in the Na layers would stabilize the diffusion channels during charge/discharge processes^44^. HAADF-STEM can thus visualize the Zn dopants located at the Na sites. We then performed ^23^Na solid-state nuclear magnetic resonance (NMR) spectroscopy for NLMO and NLMO-Zn to compare their sodium chemical environments, and the results reveal that the doped Zn^2+^ ions do not exist in the transition-metal layer (Supplementary Fig. 6)^45^. Altogether, these analyses provide strong evidences that the doped Zn preferentially occupies the Na layer rather than the transition-metal layer.

The morphologies and structures of both samples were further corroborated by scanning electron microscopy (SEM) images and elemental analyses using energy dispersive X-ray spectroscopy (EDS) and inductively coupled plasma optical atomic emission spectroscopy (ICP-AES). Both NLMO and NLMO-Zn are predominantly particles of several microns in dimensions (Supplementary Fig. 7), and all elements are homogeneously distributed in both samples (Supplementary Fig. 8). All atomic ratios are similar to their calculated values based on their crystal structures (Supplementary Fig. 9 and Supplementary Table 3, 4). Although the sodium content in Na_*x*_MnO_2_ is usually in the range of *x* = 0.68−0.76, the introduction of Li into the transition-metal layer can extend the upper limit of this range to 0.8 or 0.833^32,46^. Unfortunately, our attempts to increase the amount of Zn dopant was unsuccessful; when attempting to increase the atomic percent of Zn from 0.98% to 1.96% to obtain a stoichiometry of (Na_0.833_Zn_0.075_)[Li_0.25_Mn_0.675_]O_2_, an impure phase (i.e., LiMn_2_O_4_) emerges in its XRD pattern (Supplementary Fig. 10). Thus, substituting Zn ions for 5% Mn is found to be the optimum condition in terms of phase purity.

As an experimental indicator of thermal stability, we characterized NLMO and NLMO-Zn by TGA and DSC (Supplementary Fig. 11). Both samples exhibit two exothermic events as marked by the arrows in Supplementary Fig. 11b, but these occur at higher temperatures for NLMO-Zn than for NLMO, corroborating the computational finding that the Zn-doped compound possesses higher thermal stability as shown in Supplementary Fig. 1.

### Experimental validation of electronic and thermodynamic stability

**Fig. 5:**
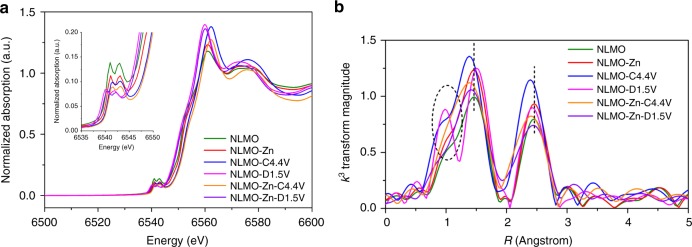
Observation of Jahn–Teller distortion in NLMO and NLMO-Zn during the first cycle. **a** Shows the XANES spectra at the Mn-K edge for NLMO and NLMO-Zn before cycling as well as in the fully charged and discharged states. **b** Shows Fourier-transformed (FT) magnitude of Mn K-edge EXAFS spectra for NLMO and NLMO-Zn before cycling as well as in the fully charged and discharged states

To verify the computational results regarding Jahn–Teller distortion experimentally, we performed ex situ X-ray adsorption near-edge structure (XANES) and extended X-ray adsorption fine structure (EXAFS) analyses for the two samples in the as-prepared, fully charged, and fully discharged states during the 1^st^ cycle. The XANES spectra at Mn K-edge region are shown in Fig. 5a, and its magnified pre-edge region is shown in its figure inset. In the pre-edge region, two peaks are observable, which is consistent with previous reports^47,48^. However, these Mn pre-edge peaks look similar for both pristine NLMO and NLMO-Zn owing to their weak intensities. After full discharge to 1.5 V, these two pre-edge peaks, as well as the main absorption edge in both samples, shift to lower energies because of the decreased oxidation state of Mn. However, two pre-edge peaks and the main edge of NLMO are positioned at lower energies than those of NLMO-Zn, which implies that the Mn in NLMO has a lower average oxidation state (i.e. closer to the Jahn–Teller active Mn^3+^), and thus NLMO would suffer more severe Jahn–Teller distortion than NLMO-Zn.

From the Fourier-transformed (FT) magnitude plot of the Mn K-edge EXAFS spectra shown in Fig. 5b, two main peaks are observable in all cases. The peak at about 1.5 Å corresponds to the single scattering paths of adjacent oxygen atoms (i.e., Mn-O), while the peak at approximately 2.5 Å is attributed to the interactions between Mn ions and their neighbouring metal ions (i.e., Mn, Zn, or Li)^46,47^. Note that these distances are not the actual bond lengths because these FT spectra were not phase-corrected. For NLMO in the fully discharged state, an additional peak at 0.98 Å (circled in dashed lines) resulting from Jahn–Teller distortion can be clearly resolved, but this peak is not observed in NLMO-Zn in the fully charged and discharged states evidencing our hypothesis and computational calculations regarding the suppression of Jahn–Teller distortion by the doped Zn.

**Fig. 6:**
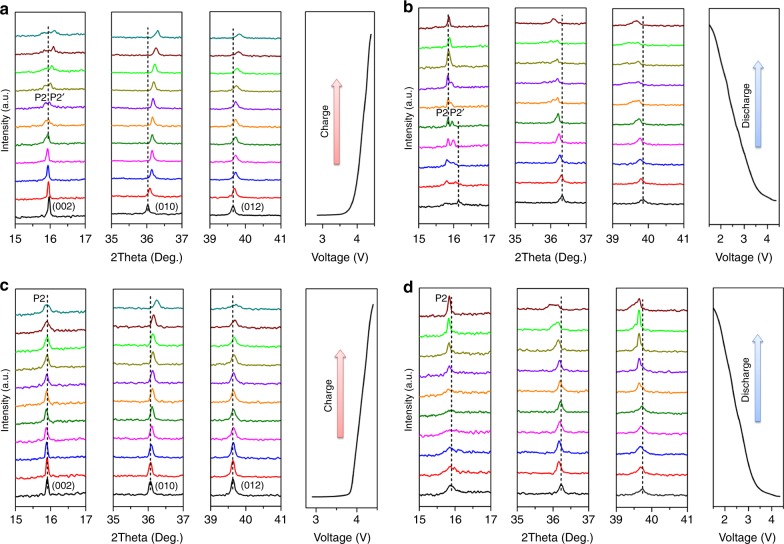
The phase changes of NLMO and NLMO-Zn during the 1st cycle. In situ XRD patterns of NLMO (**a**, **b**) and NLMO-Zn (**c**, **d**) during the 1st charge/discharge are shown

To figure out the structural changes of NLMO and NLMO-Zn during charge/discharge, in situ XRD analysis was carried out during the 1st cycle, as shown in Fig. 6. For pristine NLMO, the (002) peak splits into two peaks when the sample was charged to 4.12 V (purple line in Fig. 6a), which indicates that two phases coexisted in this high voltage region. Because the (002) peak of the O2 phase is located at around 20°, the newly emerged phase has nothing to do with the O2 phase^49^. However, any peak assigned to the O2 phase does not emerge in both samples (Supplementary Fig. 12), but the (002) peak splits into two adjacent peaks for pristine NLMO, demonstrating that a phase transformation occurs to form a new phase. Because the new phase is similar to the P2 phase in the structural viewpoint, it is named P2’ phase. During charge, the (002) peak of the P2 phase keeps decreasing gradually and finally almost vanishes in the fully charged state. The phase transformation and two-phase behaviour of pristine NLMO are induced by the phase separation stemming from thermodynamic phase instability as predicted by our first-principles calculations above. The (002) peak of the P2’ phase gradually shifts to high angles during the charging process from 4.12 V to 4.4 V, which is indicative of the decrease of layer spacing. The (010) and (012) peaks also shift to high angles during charge because of their reduced lattice parameters as well. Figure 6b shows the in situ XRD patterns of pristine NLMO during discharge. As Na ions are inserted back into interlayers, the (002) peak of P2’ phase gradually shifts to lower angles, and its intensity simultaneously decreases. Then, it completely disappears when discharged to 2.08 V. On the other hand, the (002) peak of the P2 phase reappears, and its intensity gradually becomes higher during discharge. Likewise, the (010) and (012) peaks gradually return to the original angles in the initial state during discharge.

Supplementary Fig. 13 shows how the cycling performance of pristine NLMO changes depending on the voltage window. When charged or discharged between 1.5 and 4.6 V, the P2 phase completely transforms into P2’ phase with a drastic capacity decay for the initial three cycles. Between 1.5 and 4.2 V, the P2–P2’ transition is avoided, thereby enhancing the capacity retention. However, with this condition, the initial capacities and coulombic efficiencies are relatively low. When the charge terminal voltage is increased to 4.7 V, the electrolyte is completely decomposed (Supplementary Fig. 14). Both high capacity and decent capacity retention could be achieved via the improved thermodynamic phase stability only when the charging voltage is set to 4.4 V.

Figure 6c shows the in situ XRD patterns of NLMO-Zn when charged to 4.4 V. Interestingly, any peak assigned to the P2’ phase is not detected, and the (002) peak of the P2 phase just broadens during charge, substantiating the argument that the phase transition or separation into the P2’ phase is suppressed thanks to the presence of a ground state at *x* = 0.75 in the mixing enthalpy diagram (Fig. 2b). The (010) and (012) peaks of NLMO-Zn shift in a similar manner to pristine NLMO, but less compared with the pristine sample, proving that NLMO-Zn undergoes less lattice parameter change during charge. The (002) peak of the P2 phase slightly shifts to lower angles (Fig. 6d), indicating that the interlayer spacing of NLMO-Zn is a little increased during discharge. Just like the pristine sample, the (010) and (012) peaks return back to their original positions before cycling. Hence, it is completely verified that NLMO-Zn does not undergo P2 to P2’ phase transition unlike the pristine NLMO, which well agrees with the thermodynamic phase stability results from first-principles calculation. Hence, Zn doping may well contribute to improving the cycling performance of NLMO-Zn over the undoped counterpart.

The (002) peak of the P2 phase in NLMO and NLMO-Zn was also observed after the 2nd and 100th cycles (Supplementary Fig. 15). The (002) peak of NLMO-Zn has no obvious change even after the 2nd and 100th cycles, whereas the undoped NLMO has a new peak located beside the (002) peak, which is assigned to the P2’ phase. The intensity of this peak becomes higher as the cycle number increases because the P2’ phase cannot completely revert to the P2 phase every cycle, demonstrating that the phase separation of undoped NLMO becomes more serious with the cycle number.

### Electrochemical performance

**Fig. 7:**
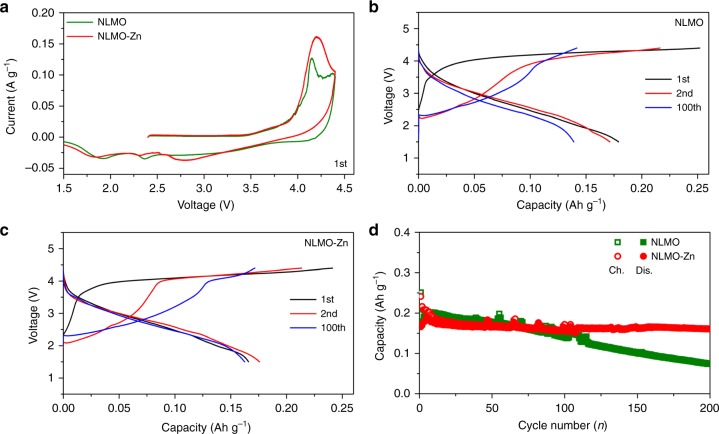
Cycling performance and electrochemical behaviours of NLMO and NLMO-Zn. CVs of NLMO and NLMO-Zn in the 1st cycle (**a**). Charge-discharge curves of NLMO (**b**) and NLMO-Zn (**c**) in the 1st, 2nd, and 100th cycle. Comparison of the cycling performance between NLMO and NLMO-Zn at 0.2 C (**d**)

Based on the computational and experimental results above regarding the reduction of the Jahn–Teller distorted Mn^3+^ centers and the improved phase stability, Zn doping must enhance the capacity retention of NLMO. We verified this by comparatively evaluating the electrochemical performances of NLMO and NLMO-Zn in coin cells using Na as the anode. In the 1st cycle of the cyclic voltammogram (CV) in Fig. 7a, NLMO exhibits two oxidative peaks at 4.15 V and 4.38 V. Herein, the peak at 4.15 V can be assigned to the oxidation of Mn^3+^ and O^2−^, while the peak at 4.38 V may be attributed to the phase transformation as previously reported^26,28,29,35^. We nonetheless also considered the possibility of anion redox-based on some recent literature findings^29,32,41^ as well as ex situ XPS characterisation for the charged or discharged electrodes (Supplementary Fig. 16). In the CV of NLMO-Zn, only one oxidation peak at 4.20 V is present, which is assigned to the oxidation of O^2−^ and Mn^3+^ ions. Herein, the absence of the second oxidation peak may indicate that this material does not undergo phase change as previously demonstrated. The reductive sweep during the 1st cycle shows the cationic reduction of Mn^4+^ as well as anionic reduction of O_2_^n−^. The redox peaks appear asymmetric because the reaction process during desodiation is different from that during sodiation in the 1st cycle as reported in previous papers^32,41^. In addition, all redox peaks have sizeable magnitude of current densities, indicating the involvement of significant amount of redox-active centers.

As shown in the charge/discharge curves of NLMO (Fig. 7b) and NLMO-Zn (Fig. 7c) for the 1st, 2nd and 100th cycle, both samples exhibit similar changes in electrochemical behaviours upon repeated cycling in spite of the apparent superiority of NLMO-Zn. The charge curves of both samples look similar during the whole cycling in that there is a sloping region between 2.2 and 3.8 V and thereafter a plateau region. Actually, as the cycle number increases, the capacity contribution of the sloping region becomes larger, whereas that of the plateau region keeps falling down. The changes are the most prominent up to the 2nd cycle, but get less from the 3rd cycle onward. These changes have been observed in previous studies as well and attributed to the activation of Mn^4+^ to Mn^3+^^32,41^. This plateau region decreases from the 1st to the 100th cycle more rapidly in NLMO than in NLMO-Zn, which is attributed to their different desodiation pathways as shown in the CV curves (Fig. 7a) and in the in situ XRD patterns. Although the charge and discharge curves of both NLMO and NLMO-Zn undergo continuous shape change as the cycling proceeds, the capacity contribution above 3.9 V is a bit higher for NLMO-Zn than for NLMO after the 1st cycle (Supplementary Fig. 17). While discharge proceeds, both samples show similar discharge curves in which the continuous slope is maintained down to the cut-off voltage, 1.5 V, but the discharge capacity of NLMO displays much faster decay than that of NLMO-Zn up to the 100th cycle.

Figure 7d shows the cycling performance of NLMO and NLMO-Zn. Because the redox-active Mn centers are partially substituted by the redox-inactive Zn dopant in NLMO-Zn, the doped sample exhibits lower initial capacity than that of NLMO. However, with repeated charge/discharge cycles, NLMO-Zn maintains very stable capacity retention, while the capacity of NLMO gradually decays up to the 60th cycle, finally rendering the capacity of NLMO-Zn to surpass that of NLMO. In the 1st cycle, the charge and discharge capacities of the doped sample respectively correspond to 0.241 Ah g^−1^ and 0.166 Ah g^−1^, which are a little lower than those of its undoped counterpart (0.252 Ah g^−1^ and 0.179 Ah g^−1^, respectively). However, NLMO-Zn exhibits a discharge capacity of 0.162 Ah g^−1^ in the 100th cycle, which is similar to its 1st cycle discharge capacity and exceeds that of NLMO that has dropped down to 0.139 Ah g^−1^. Up to the 200th cycle, NLMO-Zn still maintains the same discharge capacity reaching 0.161 Ah g^−1^, which is two times higher than that of NLMO (0.074 Ah g^−1^), and a coulombic efficiency (CE) of 100.06%, which is superior to that of the undoped sample at 98.20% (Supplementary Fig. 18). Hence, these results clearly demonstrate that Zn doping in NLMO enables superior capacity retention, which can be attributed to the suppression of Jahn–Teller distortion and phase separation as consistent with our hypothesis and computational predictions above. From the morphological observation of the electrodes before and after cycling (Supplementary Fig. 19), the particle sizes of both samples are well maintained even after 100 cycles. In addition, the reproducibility of these electrochemical results was confirmed (Supplementary Figs. 20−22).

## Discussion

**Fig. 8:**
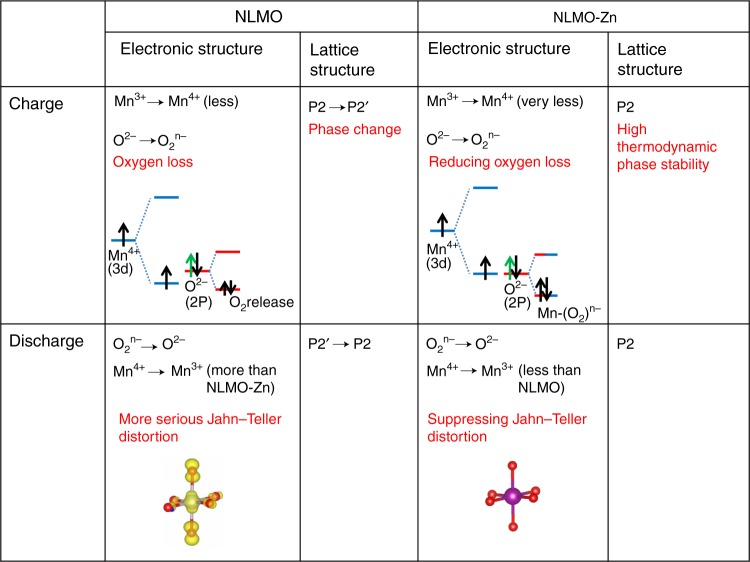
The comparative electronic and lattice structure changes between NLMO and NLMO-Zn

Figure 8 shows the changes in the electronic and crystal structures of NLMO and NLMO-Zn during the 1st cycle. The initial valences of Mn ions in pristine NLMO and NLMO-Zn are commonly close to +4, even if that of NLMO-Zn is quite higher than that of pristine NLMO, thus suppressing Jahn–Teller distortion. Hence, the charge capacities of NLMO and NLMO-Zn may significantly depend on the oxygen redox reaction (O^2−^ → O_2_^n−^), as evidenced by XPS spectra in the O 1*s* region in the fully charge/discharged states (Supplementary Fig. 16). During the 1st charge, the phase transition from P2 to P2’ takes place in NLMO, but this is not observed in NLMO-Zn. The phase transition in NLMO results from higher O 2*p* than Mn 3*d*_*t2g*_^50^, which makes an irreversible oxygen loss inevitable. During the 1st discharge, the P2’ phase reverts to the P2 phase, and the reduction reactions of O_2_^n−^ and Mn^4+^ occur simultaneously. During discharge, the phase reversion from P2’ to P2 is accompanied by the reduction of both O_2_^n−^ and Mn^4+^ in NLMO. During recharge, the reduction from Mn^4+^ to Mn^3+^ becomes higher in NLMO than in NLMO-Zn because of the oxygen loss resulting from the phase transition from P2 to P2’.

As the activation process of Mn^4+^ ions occurs for both samples, the change of Mn valence in NLMO and NLMO-Zn was also observed after 50 cycles, and the ratios of Mn^3+^ to Mn^4+^ in NLMO and NLMO-Zn are 1:7.2 and 1:15.2, respectively (Supplementary Fig. 23). The poor phase stability of NLMO leads to the phase transition from P2 to P2’ and significant oxygen loss, which is accompanied by more reduction of Mn ions, thereby continuously increasing the amount of Mn^3+^ during the repeated cycling. After 50 cycles, the differences in capacity retention between NLMO and NLMO-Zn become larger and larger with the cycle number.

Due to the difference in electronegativity, the doped Zn likely occupies the original Mn^3+^ sites in NLMO, and thereby reduces the amount of Mn^3+^ through modulating the electron density in NLMO-Zn. Considering this positive and advantageous effect of the Zn dopant, other non-transition metals were screened computationally as well to figure out whether they have similar benefits (Supplementary Fig. 24, 25). Interestingly, Mg-doped Na[Li_0.25_Mn_0.75_]O_2_ is predicted to show significantly reduced Jahn–Teller distortion because its Mn electronic structure is similar to that of Zn-doped Na[Li_0.25_Mn_0.75_]O_2_. In good agreement with our predictions, a recent paper showed the improved structural stability of Na_2/3_Mn_1−*y*_Mg_*y*_O_2_ (*y* = 0.05 and 0.1)^51^.

In addition, Zn doping significantly contributes to improving the thermodynamic phase stability of the Mn-based layered oxide. The influence of Zn doping on the electrochemical performance of other Mn-based cathode materials were also investigated, and P2-type Na_2/3_MnO_2_, O3-type NaMnO_2_, and their Zn-doped counterparts were synthesized. As demonstrated by the capacity retention results (Supplementary Fig. 26, 27), both P2-type and O3-type Mn-based cathode materials clearly exhibit enhanced cyclic retention after Zn doping.

Here, all experimental results agree well with the predictions from our first-principles calculations. Based on our empirical and computational findings, it seems that Zn doping has two positive effects on NLMO. First, Zn^2+^ would primarily results in Jahn–Teller distortion of Mn^3+^. Secondly, and Zn^2+^ doping makes the phase stability of the P2 structure robust during charge/discharge and thus helps to prevent its phase transition to the P2’ structure. Consequently, NLMO-Zn could exhibit significantly improved phase stability and capacity retention in comparison with NLMO. Even if the content of Zn in NLMO-Zn is not that high, it looks sufficient for stabilizing the crystal structure of NLMO, as similar to the previous reports with small amount of doping^31,52^. Thus, Zn doping may be an effective and broadly applicable strategy for Mn-based cathode materials.

## Methods

### Material preparations

NLMO and NLMO-Zn were prepared using a sol–gel method with a subsequent heat treatment. For NLMO-Zn, LiCH_3_COO (49.5 mg, 0.750 mmol), NaCH_3_COO (211.2 mg, 2.575 mmol), Mn(CH_3_COO)_2_∙4H_2_O (523.9 mg, 2.138 mmol) and Zn(NO_3_)_2_ (33.5 mg, 0.113 mmol) were dissolved in deionized water. Citric acid (1.73 g) and PEG400 (2 mL) were added to the solution and continuously stirred for 30 min. The transparent solution was evaporated at 80 °C for 12 h and then heated in a vacuum oven at 60 °C for 4 h, yielding a viscous white precursor. This precursor was calcined at 400 °C for 2 h and then annealed at 700 °C for 6 h in air. After cooling to room temperature, the product NLMO-Zn was obtained as a purple powder. For NLMO, the amount of Mn precursor, Mn(CH_3_COO)_2_∙4H_2_O, was changed to 551.5 mg (2.25 mmol); the Zn precursor was not added.

### Calculation methods

Densities of states and thermodynamic values were calculated using the density functional theory (DFT) and implemented using a Vienna ab initio simulation package (VASP), which was based on a plane-wave set with the pseudopotentials of a projector-augmented wave (PAW). A generalized gradient approximation (GGA) of a Perdew–Burke–Ernzerhof (PBE) was used for a functional exchange-correlation parameterization. A Li pseudopotential was treated with one 2*s* electron and two 1*s* electrons, and one 3*s* electron and six 2*p* electrons for Na, to achieve their valence states. Standard potentials were used for Mn and Zn.

To find a strong correlation between transition-metal 3*d* bands, a Hubbard-type *U* correction was used in the GGA and PBE with spin-polarized calculations. For standard computational parameters, a kinetic energy cut-off of 400 eV and a k-point mesh in a reciprocal space of 4 × 4 × 2 were applied to all calculations. For all DFT calculations, cell parameters and atomic positions were fully relaxed to achieve optimized electronic structures.

The homogeneous bulk free energy (∆*G*_hom_) was calculated by the thermodynamic mixing enthalpies using a double-well function with the enthalpy coefficient, *Ω*, as follows:^43,53^

1 $$\Delta H_{\mathrm{mix}} \approx \varOmega \left( {x - x_i} \right)^2\left( {x_f - x} \right)^2 = \Delta H_{\hom },x_i \le x \le x_f$$

where Δ*H*_hom_ indicates the homogeneous mixing enthalpy from *x*_*i*_ to *x*_*f*_. $\Delta G_{\hom }^c$ can be obtained using a theoretical model including the configurational entropy, Δ*S*_hom_, with temperature (*T*) as follows:

2 $$\begin{array}{ccccc}\\ \Delta G_{\hom } = & \Delta H_{\hom } - T\Delta S_{\hom } \approx \varOmega \left( {x - x_i} \right)^2\left( {x_f - x} \right)^2\\ \\ & + 2k_{\mathrm{B}}T_x\ln \,x + 2k_{\mathrm{B}}T\left( {1 - x} \right)\ln \left( {1 - x} \right)\\ \end{array}$$

where *k*_B_ refers to the Boltzmann constant.

### Material characterizations

The crystal structures of the samples were analyzed by powder X-ray diffraction (Rigaku Ulyima IV, Cu K_α_, operating at a current of 30 mA and a voltage of 40 kV). The morphologies and microstructures of the samples were characterised using a field emission-scanning electron microscopy (FESEM, JEOL, JSM-6700F) and a transmission-electron microscopy (TEM, JEOL, JEM-2100F). Elemental analyses were performed using energy dispersive X-ray spectrometer (EDS, X-Max^N^_,_ Oxford Instruments) and an inductively coupled plasma-optical emission spectrometer (ICP-OES, OPTIMA 8300, Perkin-Elmer). The oxidation states of the elements in the samples were characterised by XPS (Perkin Elmer PHI 1600 ESCA system). Thermogravimetric analysis (TGA) and differential scanning calorimetry (DSC) were conducted using a thermal gravimetric analyzer (TA instruments, SDT Q600) and a DSC analyzer (DSC 200 F3 Maia, NETZSCH), respectively. ^23^Na solid-state nuclear magnetic resonance (NMR) experiments were performed at 132.3 MHz on a Bruker Avance II spectrometer using a 4 mm MAS probe at rotor spinning speed of 60 kHz.

HAADF-STEM images were collected by a transmission electron microscope (JEM-2100F, JEOL, Japan) at 200 kV with spherical-aberration correction (CEOS GmbH, Germany). The powder was pre-treated by grinding and ion-milling and was then transferred onto a TEM grid. HAADF-STEM image simulations were performed via QSTEM [Christioph Koch, Determination of Core Structure Periodicity and Point Defect Density along Dislocations, PhD thesis, Arizona State University (2002)].

Mn-K edge X-ray adsorption near edge structure (XANES) spectra of the samples were collected at the 10 C beam line at Pohang Accelerator Laboratory (PAL) in Pohang, South Korea,. All spectra were normalized to the main-edge jump. Additionally, high-resolution powder diffraction (HRPD) patterns were collected at National Synchrotron Radiation Research Center (NSRRC) in Taiwan. Synchrotron X-ray was monochromated to a wavelength of 0.7749 Å using a double-crystal Si (111) monochromator. The Rietveld refinement was performed by utilizing the GSAS software^54,55^.

In situ synchrotron powder XRD data were recorded in a powder diffraction beamline at the Australian Synchrotron with a wavelength of 0.6888 Å. The wavelength was calibrated according to the standard reference material (LaB_6_ 660b) of the National Institute of Standards and Technology (NIST). The cell used for the data collection was charged at a rate of 0.3 C, and the voltage window was 1.5 V–4.4 V. All in-situ XRD patterns were transferred based on Cu K_α_ standard.

### Electrochemical characterizations

All electrochemical tests were conducted in CR2032 coin-type cells. The working electrode consisted of 80 wt.% active materials, 10 wt.% conductive carbon (super P) and 10 wt.% polyvinylidene fluoride (PVdF) binder. The mixture was dispersed in N-methyl pyrrolidone (NMP) and then hand-ground for 30 min. The slurry was cast onto a Cu foil and dried at 110 °C for 12 h under vacuum. The loading of the undoped NLMO and NLMO-Zn was between 1.9 mg cm^−2^ and 3.2 mg cm^−2^. The coin cells were assembled using glass-fibre filter paper as separator, Na disc as both counter and reference electrode, and 80 μL of 1 M NaPF_6_ in ethylene carbonate/diethyl carbonate (EC/DEC, 1:1 v/v) as electrolyte. All cell assembly was performed in an argon-filled glove box. Galvanostatic charge/discharge tests and cyclic voltammetry (CV) were conducted using a WBCS battery cycler (WonATech). For charge/discharge tests, 0.25 A g^−1^ was used as 1 C.

## Electronic supplementary material

Supplementary Information

Source Data

## Data Availability

The data that support the findings of this study are available from the corresponding author upon request.
