# Supplementary material for: Manganese based layered oxides with modulated electronic and thermodynamic properties for sodium ion batteries
Source: Nat Commun. 2019 Jan 7;10:5203. doi: 10.1038/s41467-018-07646-4 (PMC6323141; doi:10.1038/s41467-018-07646-4)
Supplement: Supplementary file 1 — Supplementary Information [file 41467_2018_7646_MOESM1_ESM.pdf]

Manganese based layered oxides with modulated electronic and thermodynamic properties for sodium ion batteries

Zhang et al.

Supplementary Figures

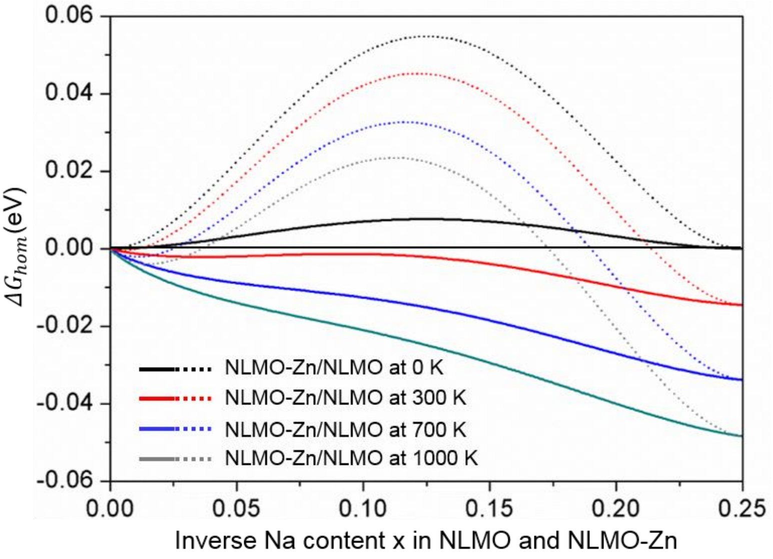

Supplementary Figure 1. Homogeneous bulk free energies as a function of the inverse Na content x in NLMO and NLMO-Zn at 0, 300, 700, and 1000 K.

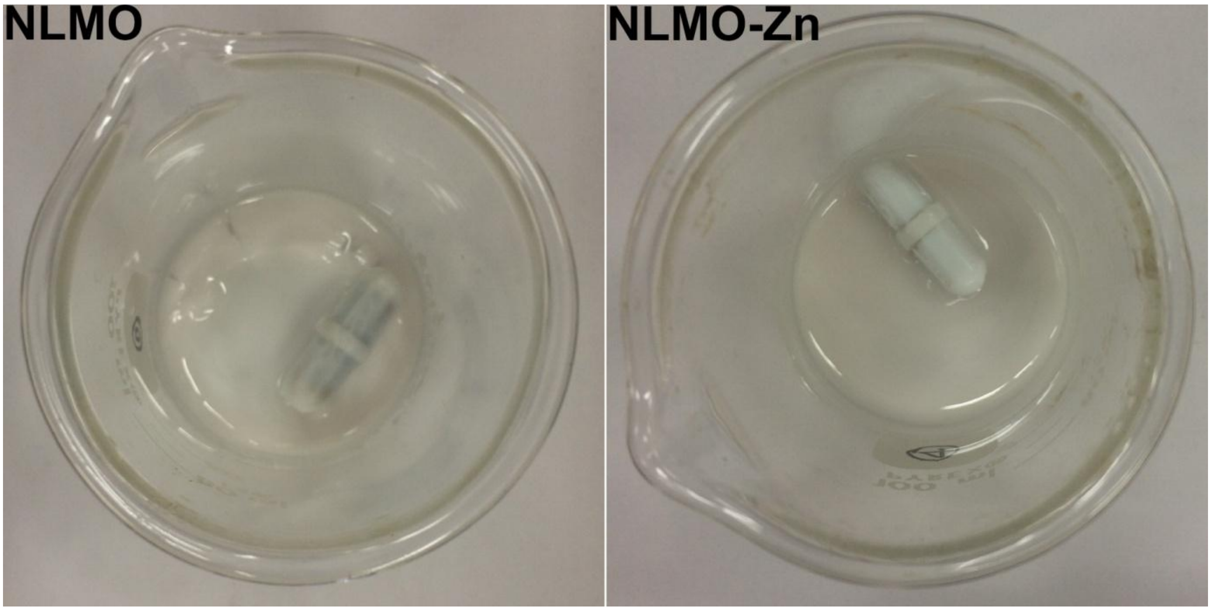

Supplementary Figure 2. Photographs of the sol-gel mixture of NLMO and NLMO-Zn after evaporation.

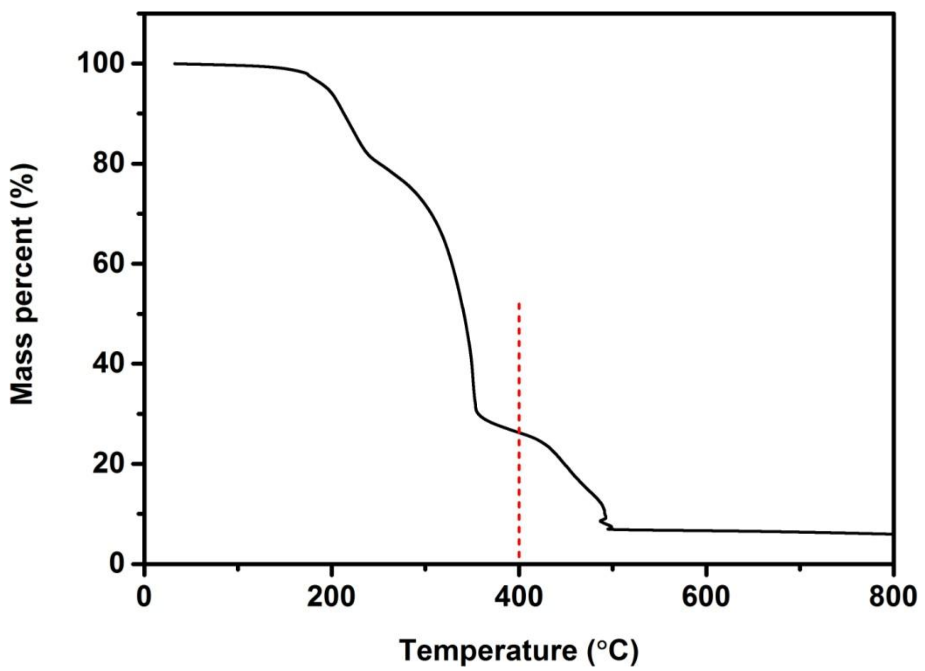

Supplementary Figure 3. TGA profile of the sol-gel mixture. 400 °C was chosen as pre-calcining temperature.

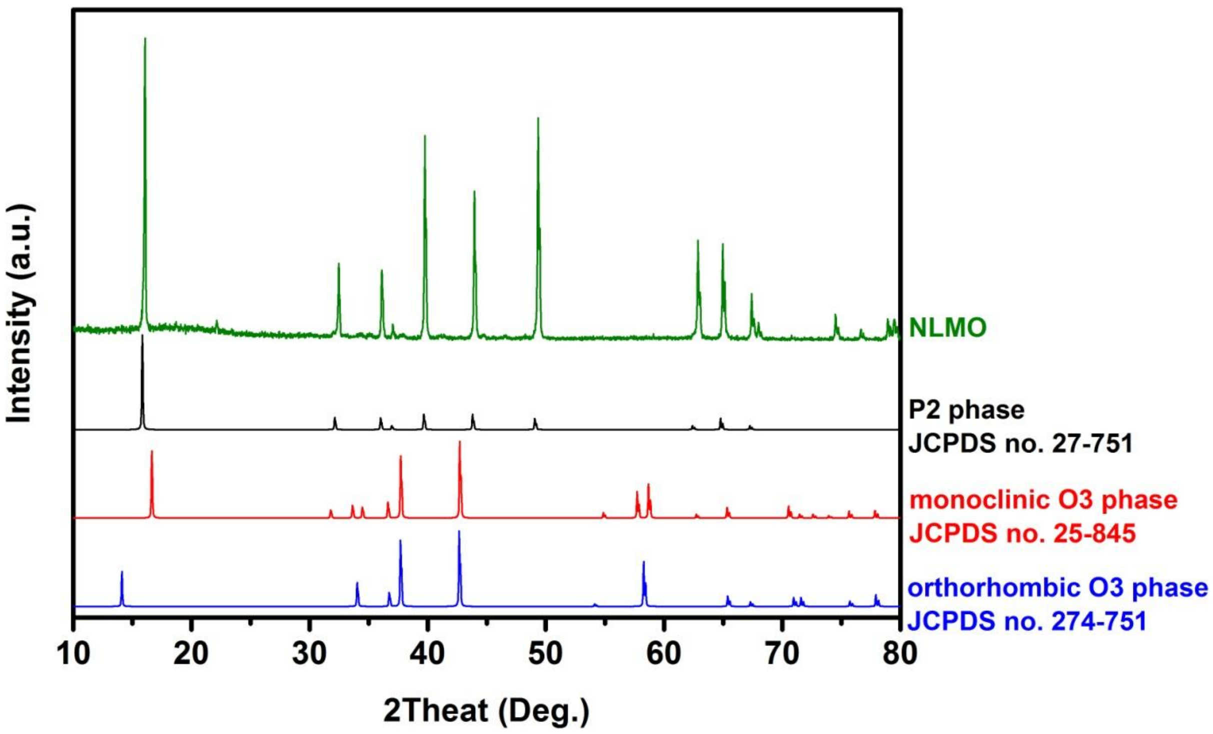

Supplementary Figure 4. XRD pattern of NLMO compared with standard JCPDS patterns.

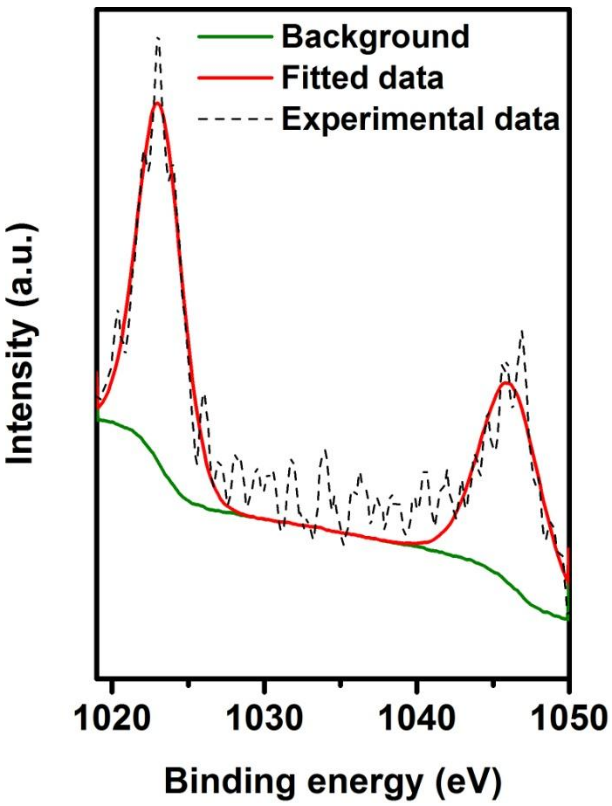

Supplementary Figure 5. Fitted XPS spectrum of NLMO-Zn in the Zn 2*p* region.

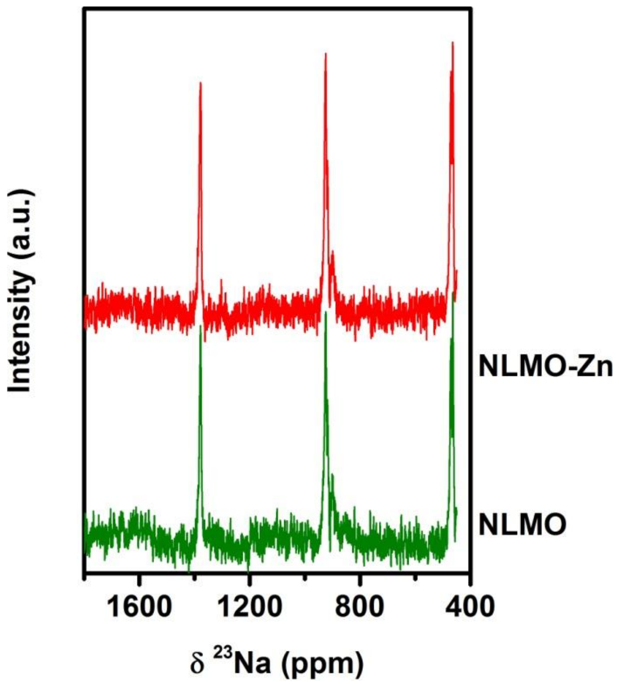

Supplementary Figure 6. ^{23}Na solid state NMR spectra of NLMO and NLMO-Zn. The main resonances of both samples are positioned between 400 and 1400 ppm. If the doped Zn^{2+} without unpaired electrons occupies the transition-metal layer to replace Mn^{3+} with four unpaired electrons, the peak should move to lower chemical shift after doping based on a previous report.[45] However, there is no obvious difference between the two samples, which means that the doped Zn^{2+} ions do not exist in the transition-metal layer.

a NLMO **b NLMO-Zn**

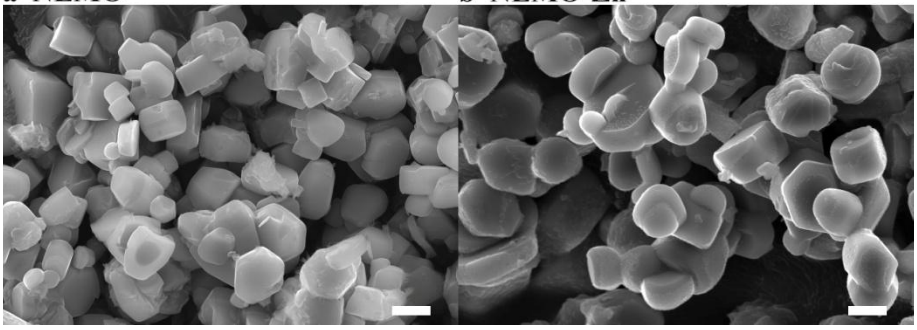

Supplementary Figure 7. Morphology analyses of NLMO and NLMO-Zn. SEM images of NLMO (a) and NLMO-Zn (b). The white scale bars represent 2 μm.

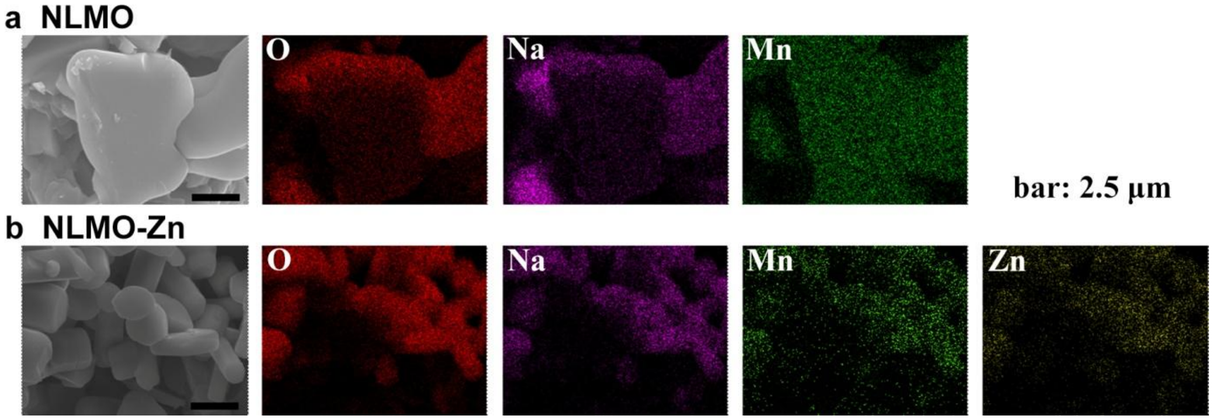

Supplementary Figure 8. Elemental distribution analyses of NLMO and NLMO-Zn. SEM images and the corresponding EDS color mapping of NLMO (a) and NLMO-Zn (b).

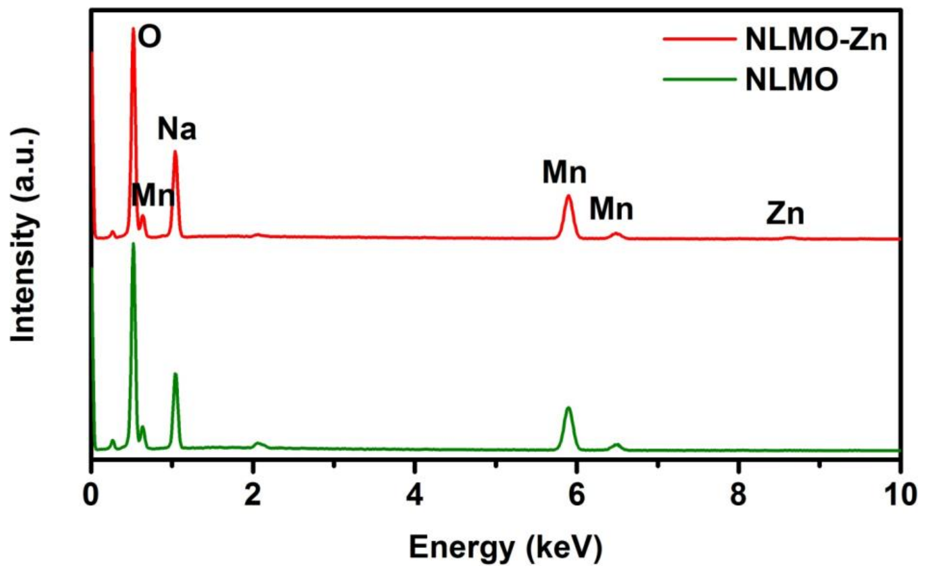

Supplementary Figure 9. EDS spectra of NLMO and NLMO-Zn.

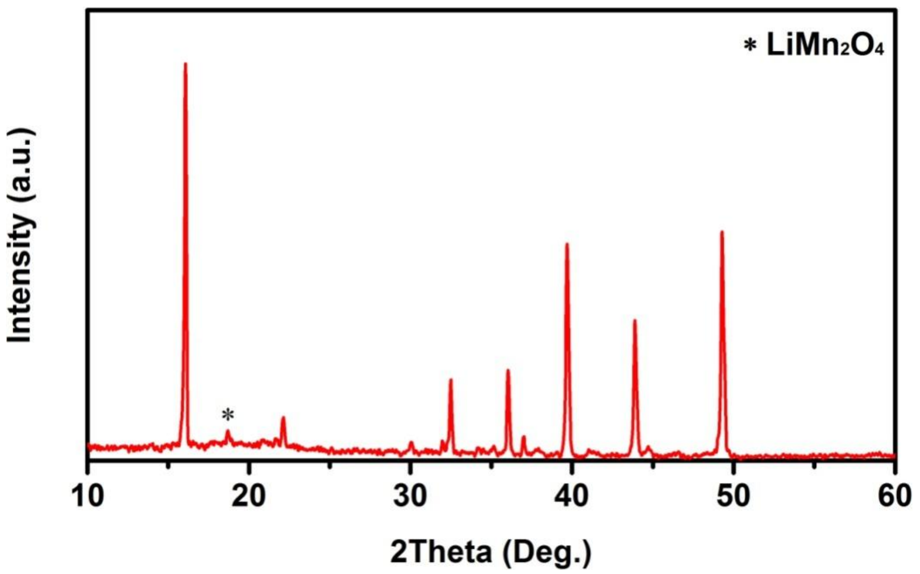

Supplementary Figure 10. XRD pattern of $(Na_{0.833}Zn_{0.075})[Li_{0.25}Mn_{0.675}]O_2$ sample.

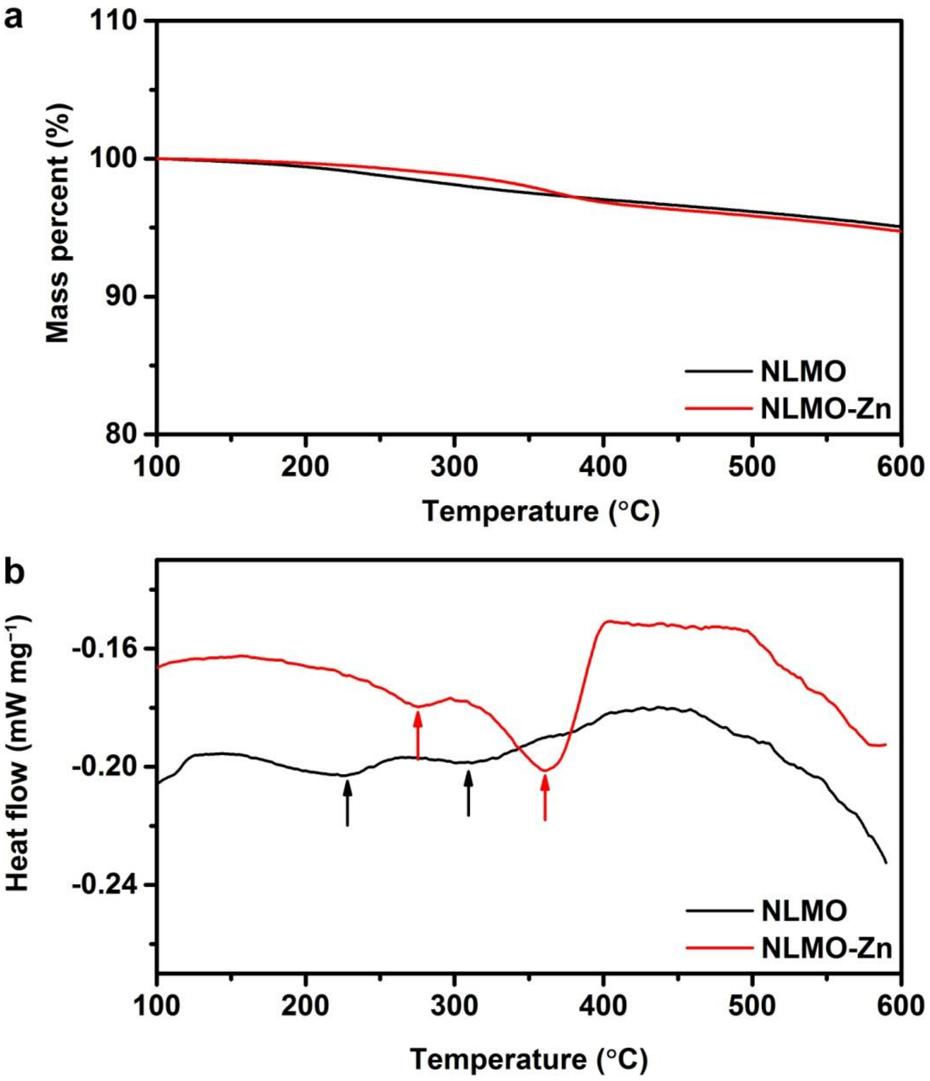

Supplementary Figure 11. Thermal stability analyses of NLMO and NLMO-Zn. TGA (a) and DSC (b) profiles of NLMO and NLMO-Zn.

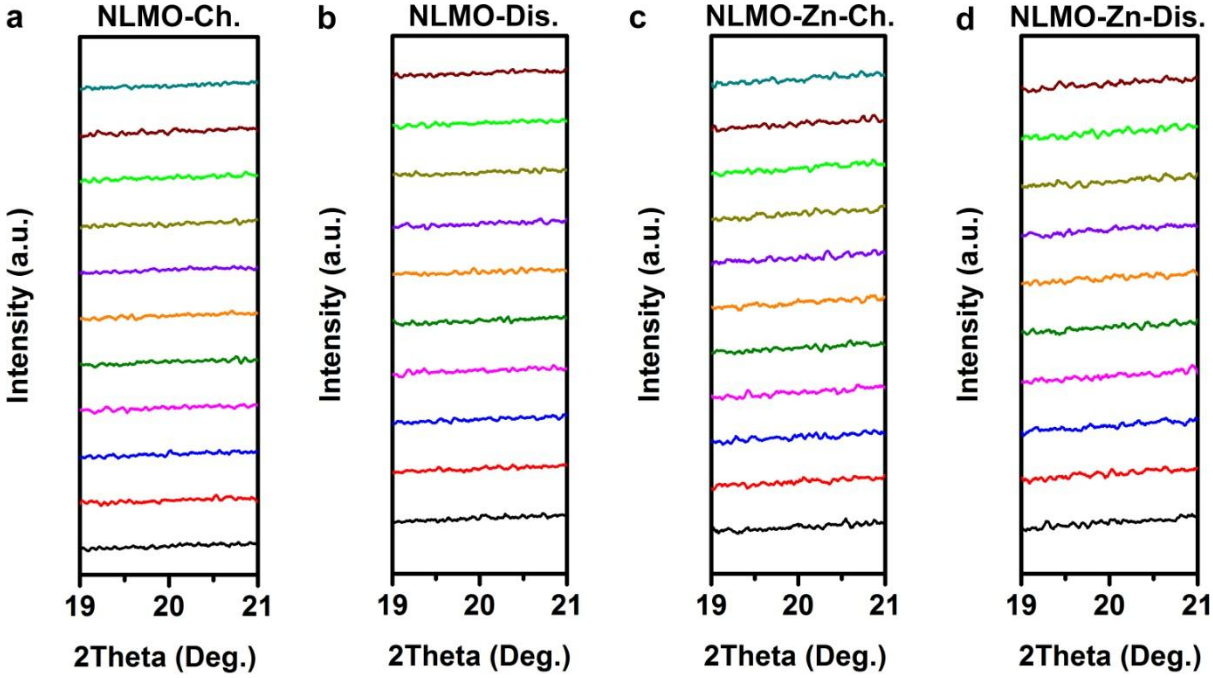

Supplementary Figure 12. Phase changes observed at in-situ XRD patterns of NLMO and NLMO-Zn when the 2θ value is between 19°–21° during the 1st cycle.

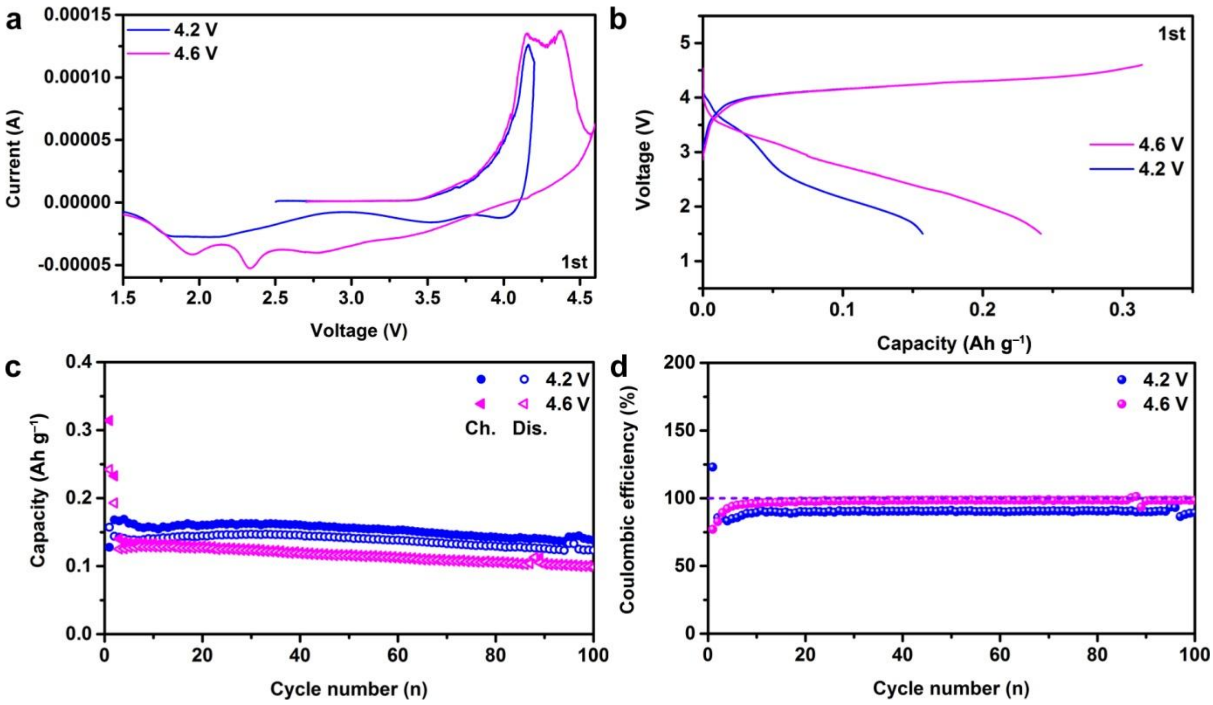

Supplementary Figure 13. Electrochemical performance comparison of NLMO when cycling at different voltage windows. CV curves (a), charge-discharge curves (b), cycling performance (c), and coulombic efficiency (d) of NLMO when using different charge terminal voltages of 4.2 and 4.6 V. When charged or discharged between 1.5 and 4.6 V, the P2 phase completely transforms into P2' phase with a drastic capacity decay from 0.242 Ah g^{-1} to 0.125 Ah g^{-1} for the initial three cycles. Between 1.5 and 4.2 V, the P2-P2' transition is avoided, thereby enhancing the capacity retention. However, with this condition, the initial capacity is just 0.157 Ah g^{-1}, and the coulombic efficiencies are less than 93% continuously from the 2nd cycle to the 100th cycle.

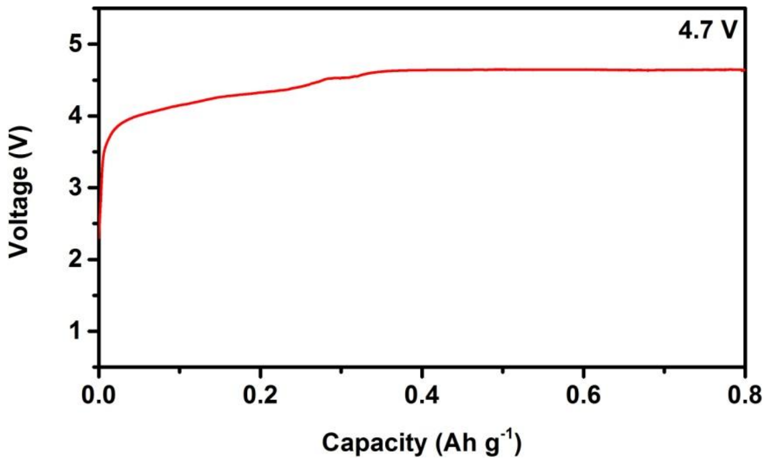

Supplementary Figure 14. Charge curve of NLMO when charged up to 4.7 V.

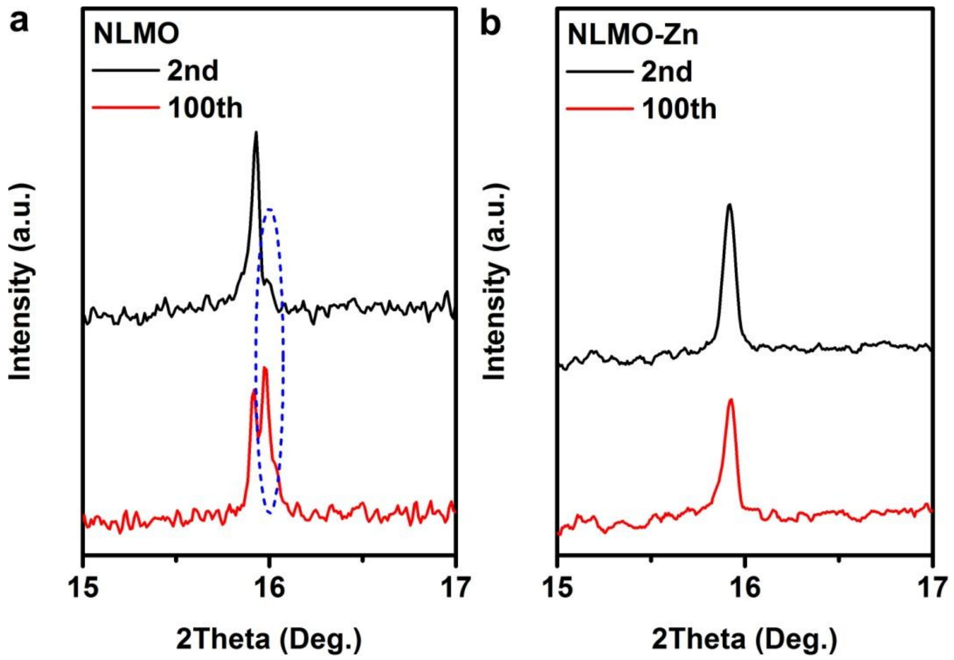

Supplementary Figure 15. Phase changes of NLMO and NLMO-Zn after 2 and 100 cycles. XRD patterns of NLMO (a) and NLMO-Zn (b) between 15−17° after the 2nd and 100th cycle.

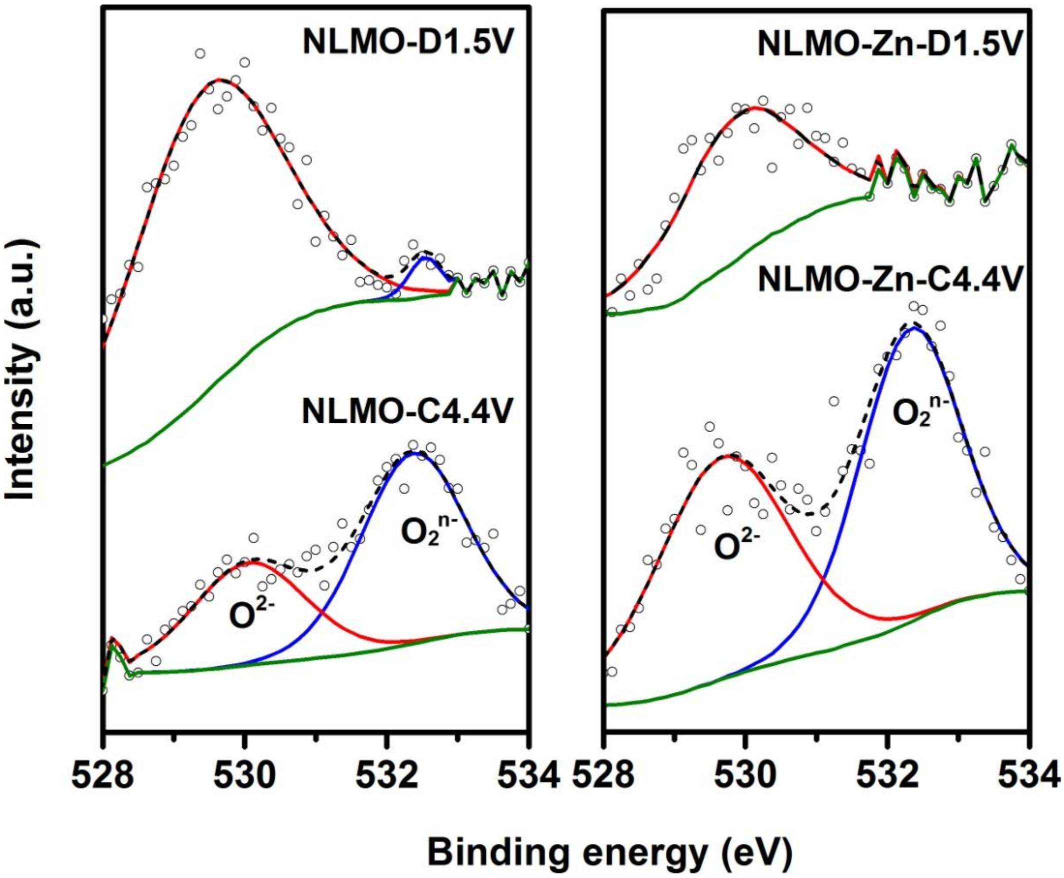

Supplementary Figure 16. XPS spectra in the O 1*s* region of the NLMO and NLMO-Zn electrodes at fully charged and discharged states during the 1st cycle. The O 1*s* partial XPS spectra show the emergence of a peak at around 532 eV for both samples upon charging to 4.4 V during the 1st cycle. This peak is assigned to an O_2^{n-} species formed from the oxidation of the oxoanion (i.e. $O^{2-} \rightarrow O_2^{n-} + (2-n)e^{-}$), indicating that the oxoanion is redox-active during charge/discharge and contributes to the capacities.

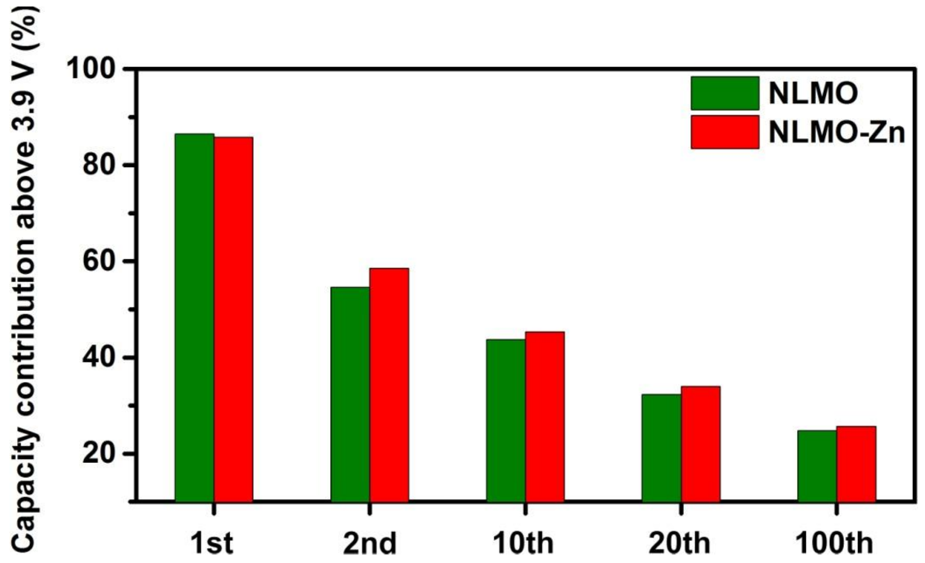

Supplementary Figure 17. Capacity contributions above 3.9 V of NLMO and NLMO-Zn at the 1st, 2nd, 10th, 20th, and 100th cycle.

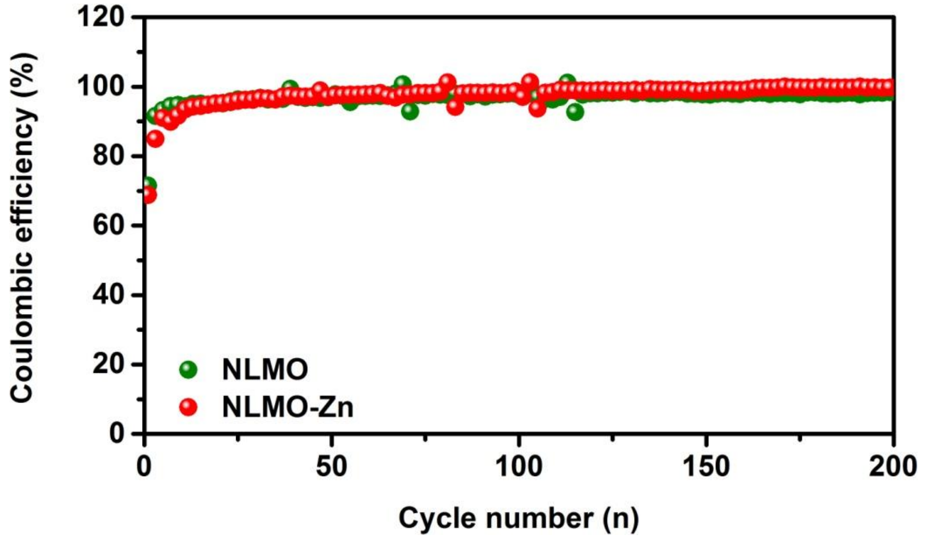

Supplementary Figure 18. Comparison of Coulombic efficiency between NLMO and NLMO-Zn at 0.2 C.

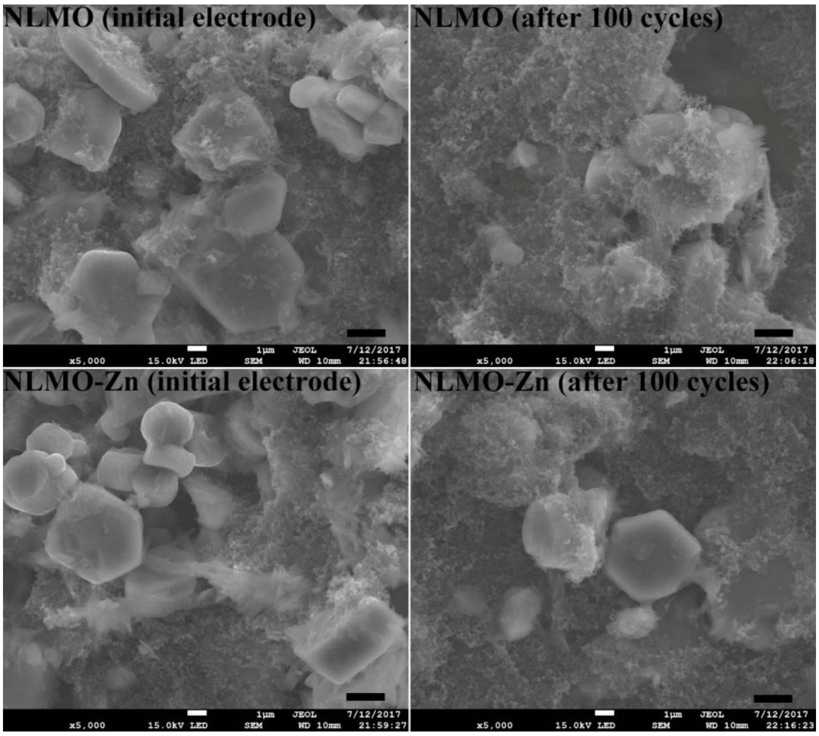

Supplementary Figure 19. Morphology changes of NLMO and NLMO-Zn electrodes during cycles. SEM images of the initial NLMO and NLMO-Zn electrodes and their electrodes after 100 cycles. Black scale bar: 2 μm.

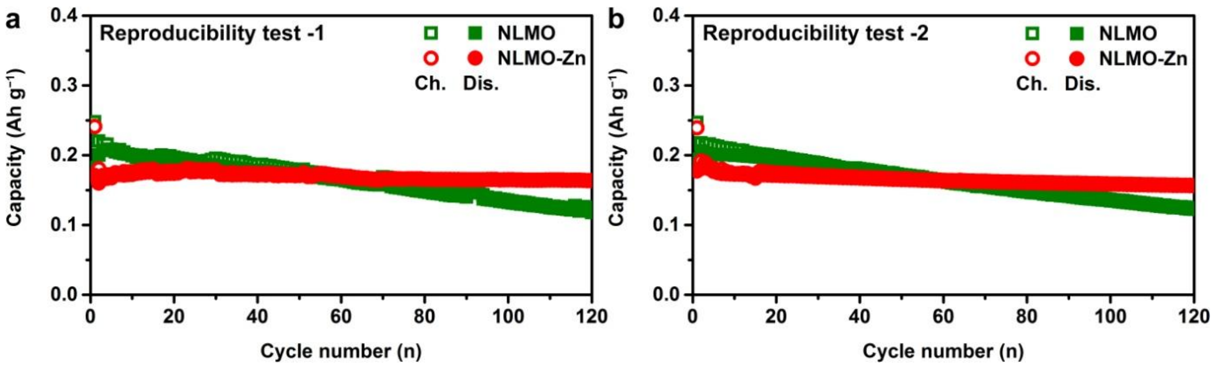

Supplementary Figure 20. Reproducibility tests for NLMO and NLMO-Zn. The data were obtained from different batches of samples.

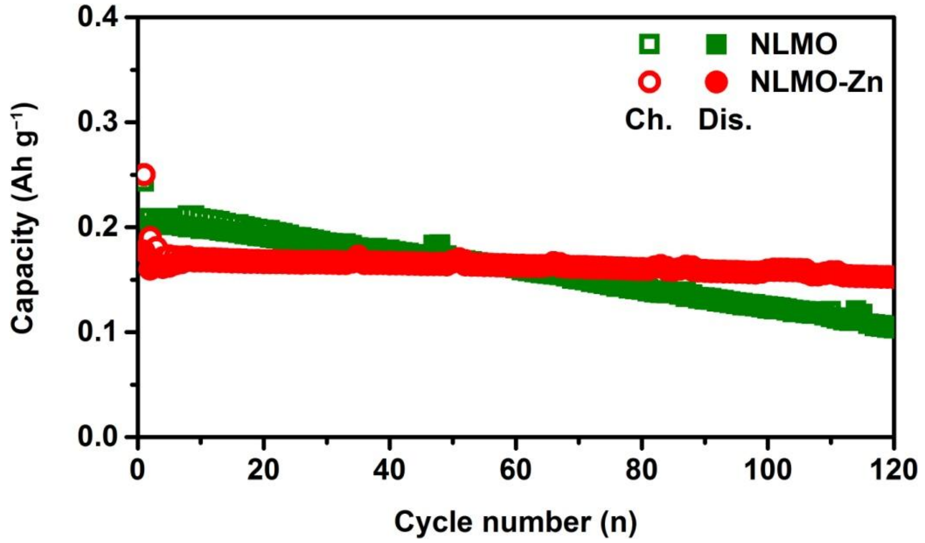

Supplementary Figure 21. Reproducibility tests for NLMO and NLMO-Zn. The samples are identical to those shown in Supplementary Fig. 20a, but there was a half year interval between cell assembly and electrochemical testing.

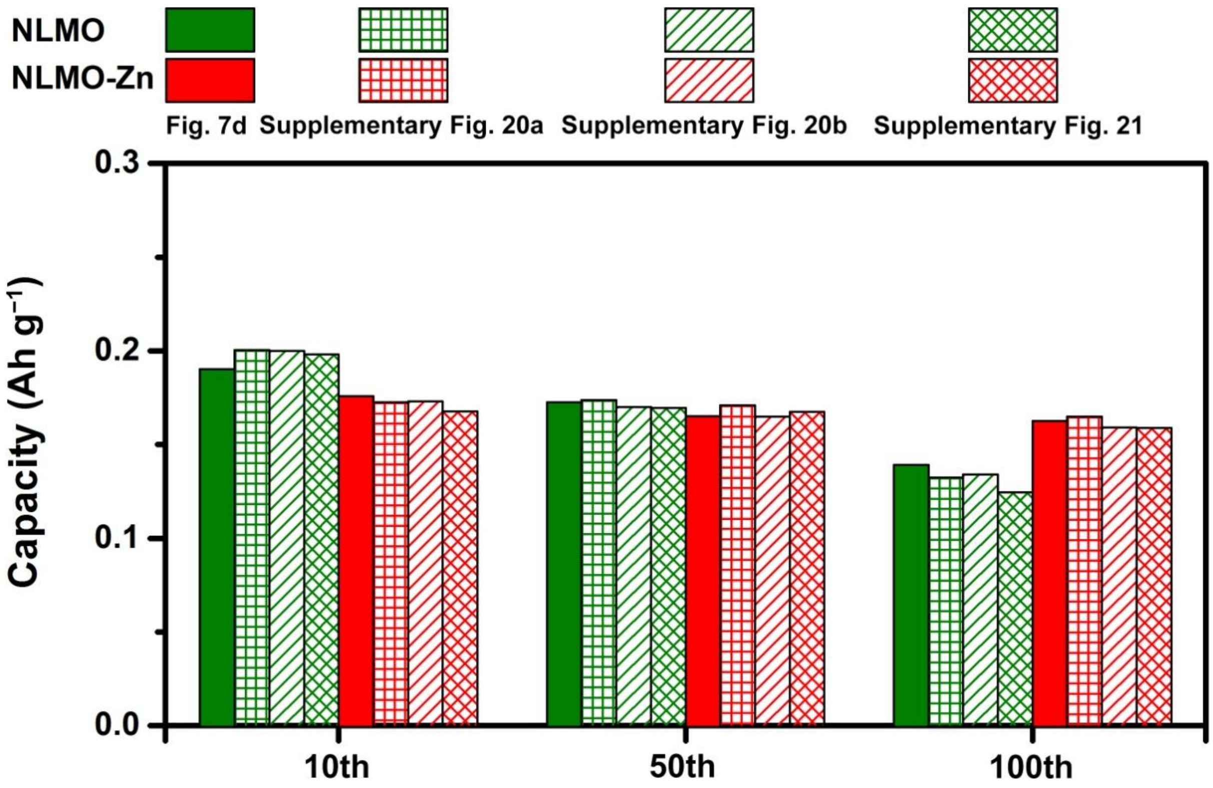

Supplementary Figure 22. Reproducibility tests for NLMO and NLMO-Zn. The discharge capacities shown in Fig. 7d, Supplementary Fig. 20a, Supplementary Fig. 20b, and Supplementary Fig. 21 were compared for doped and undoped NLMO with different cycle number. All results show almost identical electrochemical behaviours to that in Fig. 7d, resultantly confirming that the improved capacity retention in NLMO-Zn compared to NLMO is reproducible.

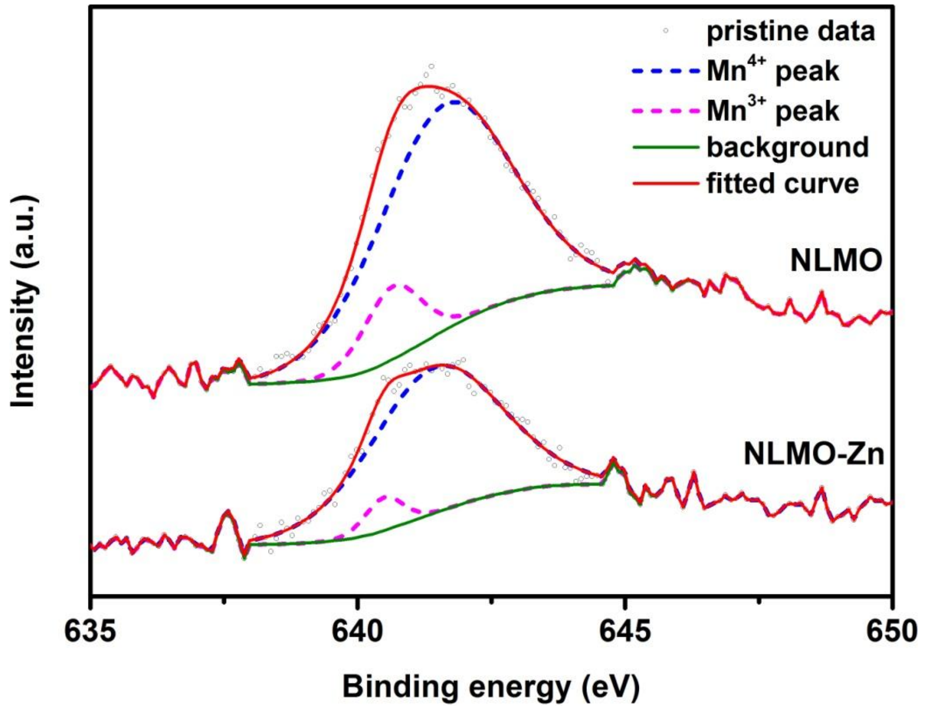

Supplementary Figure 23. XPS spectra in the Mn 2*p* region of NLMO and NLMO-Zn electrodes after 50 cycles. The ratios of Mn^{3+} to Mn^{4+} in NLMO and NLMO-Zn after 50 cycles are 1:7.2 and 1:15.2, respectively. The poor phase stability of NLMO leads to the phase transition from P2 to P2' and significant oxygen loss, which is accompanied by more reduction of Mn ions, thereby continuously increasing the amount of Mn^{3+} during the repeated cycling. The increase of Mn^{3+} definitely results in Jahn-Teller distortion, which is sure to aggravate the crystal structure of NLMO and causes continuous capacity fading during cycling. Meanwhile, NLMO-Zn maintains very stable capacity retention for more than 200 cycles. Because the amount of Zn is small in the doped sample, the capacity differences between NLMO and NLMO-Zn for the initial 40 cycles are not that large. However, the structural damage of NLMO gradually accumulates as the cycling proceeds. After 50 cycles, the differences in capacity retention between NLMO and NLMO-Zn become larger and larger with the cycle number.

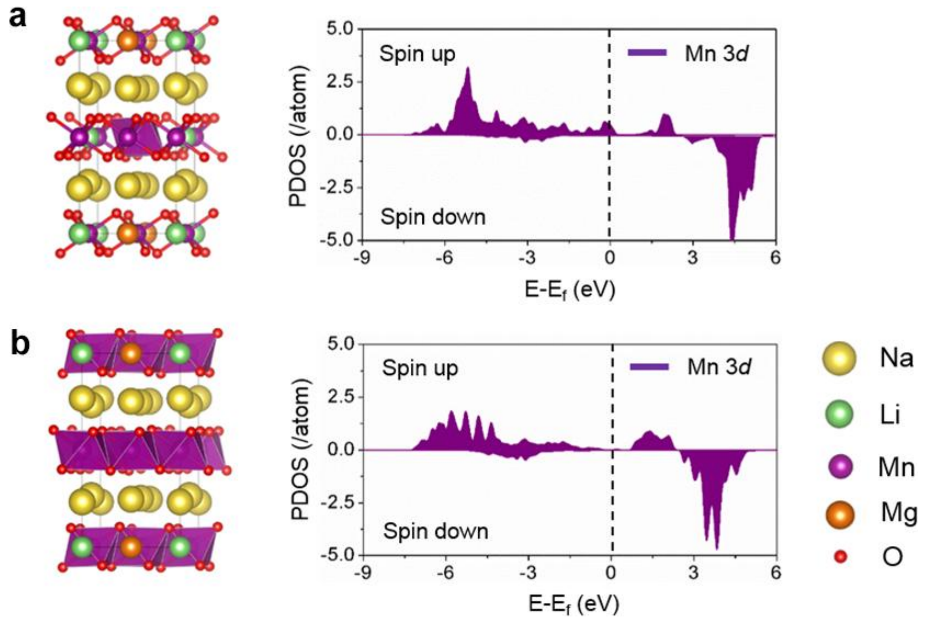

Supplementary Figure 24. Theoretical calculations regarding the effects of Mg doping in the fully sodiated Na[$Li_{0.25}Mn_{0.75}$]O_2. Partial density of states (PDOSs) of (**a**) Mn^{3+} and (**b**) Mn^{4+} 3*d* orbital electrons in the fully sodiated Mg-doped Na[$Li_{0.25}Mn_{0.75}$]O_2. These electronic structures are smiliar to the PDOSs of Mn in Fig. 1 in that the Jahn-Teller distortion is expected to be suppressed by Mg doping, which would be helpful to explain the role of Mg in the experimental results.[1]

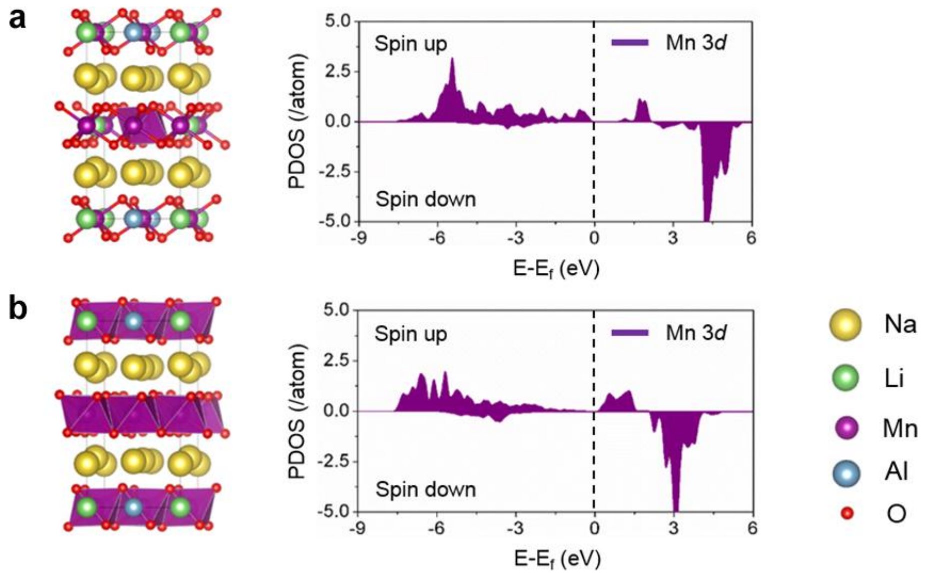

Supplementary Figure 25. Theoretical calculations regarding the effects of Al doping in the fully sodiated $Na[Li_{0.25}Mn_{0.75}]O_2$. PDOSs of (**a**) Mn^{3+} and (**b**) Mn^{4+} 3*d* orbital electrons in the fully sodiated Al-doped $Na[Li_{0.25}Mn_{0.75}]O_2$. These electronic structures for NLMO-Al are almost the same as the electronic structures of Mn 3*d*-electron in $Na[Li_{0.25}Mn_{0.75}]O_2$, (see Figs. 1a and 1b), implying that the spin-up electron of Mn in the e_g band is not interacting with the Al. While an average net charge of O in $Na[Li_{0.25}Mn_{0.75}]O_2$ is −1.2665, that of O in NLMO-Al is −1.3696. In light of the net charge calculations, we can predict that the more abundant electron density of O for NLMO-Al strengthens the bonds between the transition metals and O, which is considered to enhance the cycle stability of Al-doped Mn-based transitional metal layered oxide materials, as previously reported.[2]

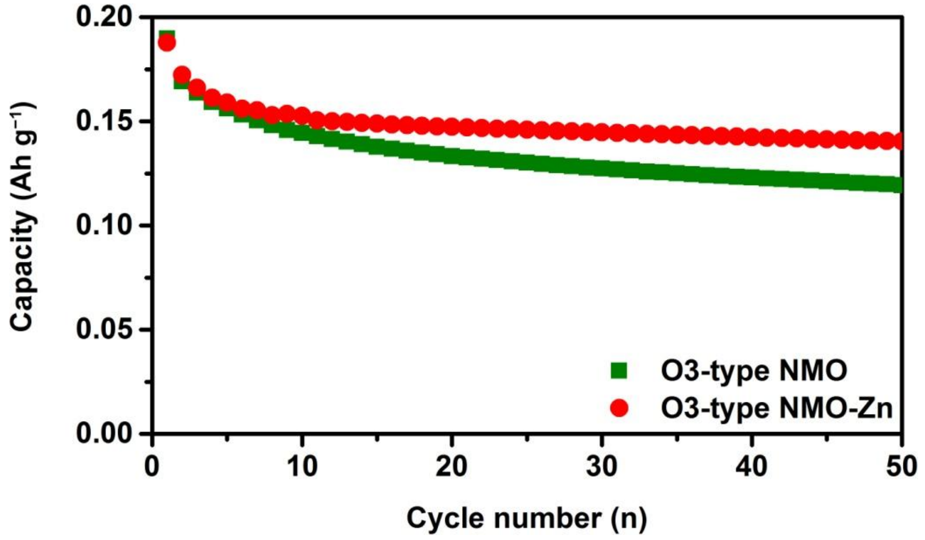

Supplementary Figure 26. Specific discharge capacities for O3-type $NaMnO_2$ (NMO) and Zn-doped $NaMnO_2$ (NMO-Zn). O3-type NMO and NMO-Zn were prepared using a solid-state method according to a previous report.[3] For O3-type NMO-Zn, Na_2CO_3 (1.219 g), Mn_2O_3 (1.500 g) and ZnO (0.081 g) were mixed. The mixture was calcined at 950 °C for 24 h with a temperature ramp of 1 °C min^{-1} in air. After cooling down to room temperature, the mixture was annealed at 950 °C for 24 h with a temperature ramp of 5 °C min^{-1} in air again. For O3-type NMO, the Mn salt amount was changed to 1.579 g. Zn salt was not added. The discharge capacities of the O3-type NMO and the O3-type NMO-Zn in the 1st cycle are 0.190 Ah g^{-1} and 0.188 Ah g^{-1}, respectively. After 50 cycles, the capacity is higher for the O3-type NMO-Zn (0.140 Ah g^{-1}) than for the O3-type NMO (0.119 Ah g^{-1}), which indicates that the cyclic retention of O3-type NMO-Zn is more enhanced compared to the O3-type NMO.

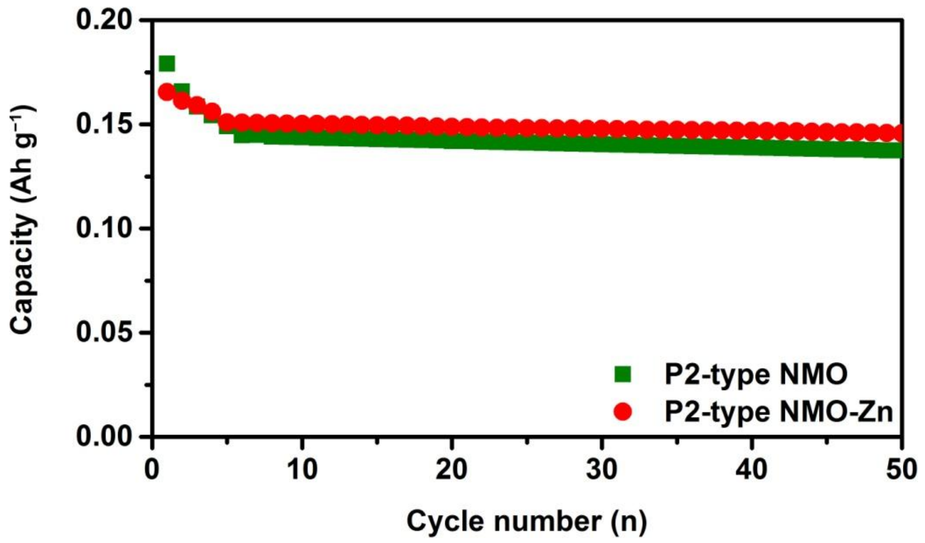

Supplementary Figure 27. Specific discharge capacities and cyclic retentions for P2-type $Na_{2/3}MnO_2$ (NMO) and Zn-doped $Na_{2/3}MnO_2$ (NMO-Zn). P2-type NMO and NMO-Zn were prepared using a liquid-state method combined with subsequent heat treatment according to a previous report.[2] For P2-type NMO-Zn, $NaNO_3$ (0.892 g), $Mn(CH_3COO)_2{\cdot}4H_2O$ (3.493 g) and $Zn(NO_3){\cdot}6H_2O$ (0.223 g) were dissolved in 10 mL of deionized water. Then, the mixture solution was stirred for 24 h at 60 °C to remove the water. The solid precursor was dried in a vacuum oven for 24 h at 80 °C. The dried precursor was calcined at 400 °C for 5 h and was then annealed at 750 °C for 12 h in air. For P2-type NMO, the Mn salt amount was changed to 3.676 g. Zn salt was not added. The discharge capacities of the P2-type NMO and the P2-type NMO-Zn in the 1st cycle are 0.179 Ah g^{-1} and 0.165 Ah g^{-1}, respectively. After 50 cycles, the capacity is higher for the P2-type NMO-Zn (0.146 Ah g^{-1}) than for the P2-type NMO (0.137 Ah g^{-1}), which indicates that the cycling performance of P2-type NMO-Zn is better than that of the P2-type NMO, and also that it performs better than the O3-type NMO. However, P2-type NMO and O3-type NMO show worse performance than P2-type NLMO.

Supplementary Note

Figs. 3b and 3c each show the Rietveld refinement results for pristine NLMO and NLMO-Zn, and the detailed cell parameters for NLMO and NLMO-Zn are listed in Table 1 and Supplementary Table 1, 2. NLMO and NLMO-Zn have a hexagonal structure in the $P6_3$ space group; the strongest peak of the two samples belongs to the (002) plane, which corresponds to the layer spacing of the P2 phase. A superlattice plane "1/3 1/3 1" is observed in both samples, indicating that the sum of the lithium and manganese ions occupying the triangular sites with $\sqrt{3}a \times \sqrt{3}a$ -type superlattice lattice is 1/3. Because of the similar ionic radius of Mn^{4+} (0.53 Å), Li^{+} (0.76 Å) and Mn^{3+} (0.645 Å), they can occupy the same crystallographic sites. In a unit cell, the lithium and manganese ions collectively occupy three different octahedral sites; one is 2a, and the other two are 2b sites. It is found that Li^{+} ions in NLMO are located in both sites, and the occupancies in the 2a and 2b sites are 0.48 and 0.12, respectively. In addition, Na^{+} ions occupy the 6c sites in which ~62.5% of Na^{+} ions share the edge and the other ~37.5% of them share the face. The fitted isotropic displacement parameters of Na ions are 4.63 $Å^2$. In the case of NLMO-Zn, Zn also occupies the 6c sites in the Na layer. The isotropic displacement parameters of Na ions in NLMO-Zn are increased to 7.94 $Å^2$. However, the crystal structure of NLMO-Zn does not change significantly compared with NLMO except for a slight cell-volume expansion from 236.43 to 238.32 $Å^3$. The corresponding P2-type crystal structure of NLMO or NLMO-Zn is shown in Figs. 3d and 3e. When viewed along the y axis, the layered structure of P2-NLMO where O-layers are stacked in ABBAAB sequence is clearly visible. Therein, Li and Mn ions occupy the same positions (2a and 2b sites) but with different occupancies. The 2a sites are marked by red circles, and the 2b positions are pointed out by using olivine circles in Fig. 3e. Each Li ion and Mn ion coordinate to six O ions to form an octahedron, and each Na ion is surrounded by six O ions to form a trigonal prism. When viewed along the z axis, some Na ions seem to surround O ions, while other Na ions are observed to surround Li and Mn ions. However, it should be noted that Na ions do not actually surround O, Li, and Mn ions due to their different z values. The occupancy of Na ions around Li and Mn ions is approximately 1/9, while that of Na ions around O ions is approximately 1/6. After doping Zn, Zn ions occupy 6c positions in the Na layer.

Supplementary Table 1. Atomic coordinates and possible occupancies of the unit cell of NLMO based on Rietveld refinement.

	site	x	y	z	g (Occupancy)	B [Å^2]
**Na1**	6c	0.730(2)	0.397(2)	0.25	0.115(1)	4.63(9)
**Na2**	6c	0.064(2)	0.064(2)	0.25	0.115(1)	4.63(9)
**Na3**	6c	0.397(2)	0.730(2)	0.25	0.115(1)	4.63(9)
**Na4**	6c	0.607(2)	0.607(2)	0.25	0.172(0)	4.63(9)
**Na5**	6c	0.728(1)	2/3	0.25	0.172(0)	4.63(9)
**Na6**	6c	2/3	0.728(1)	0.25	0.172(0)	4.63(9)
**Li1**	2b	2/3	1/3	0	0.355(7)	0.42(2)
**Mn1**	2b	2/3	1/3	0	0.644(3)	0.42(2)
**Li2**	2b	1/3	2/3	0	0.093(9)	0.42(2)
**Mn2**	2b	1/3	2/3	0	0.907(1)	0.42(2)
**Li3**	2a	0	0	0	0.300(7)	0.42(2)
**Mn3**	2a	0	0	0	0.699(3)	0.42(2)
**O1**	6c	0.018(5)	0.698(8)	0.088(7)	1	0.1
**O2**	6c	0.315(3)	0.990(7)	0.906(9)	1	0.1

Supplementary Table 2. Atomic coordinates and possible occupancies of the unit cell of NLMO-Zn based on Rietveld refinement.

	site	x	y	z	g (Occupancy, F)	B [Å^2]
**Na1**	6c	0.730(2)	0.397(2)	0.25	0.115(1)	7.94(9)
**Na2**	6c	0.064(2)	0.064(2)	0.25	0.115(1)	7.94(9)
**Na3**	6c	0.397(2)	0.730(2)	0.25	0.115(1)	7.94(9)
**Na4**	6c	0.597(7)	0.597(7)	0.25	0.172(0)	7.94(9)
**Na5**	6c	0.728(1)	2/3	0.25	0.172(0)	7.94(9)
**Na6**	6c	2/3	0.728(1)	0.25	0.172(0)	7.94(9)
**Zn1**	6c	0.730(2)	0.397(2)	0.25	0.005(0)	7.94(9)
**Zn2**	6c	0.064(2)	0.064(2)	0.25	0.005(0)	7.94(9)
**Zn3**	6c	0.397(2)	0.730(2)	0.25	0.005(0)	7.94(9)
**Zn4**	6c	0.597(7)	0.597(7)	0.25	0.007(5)	7.94(9)
**Zn5**	6c	0.728(1)	2/3	0.25	0.007(5)	7.94(9)
**Zn6**	6c	2/3	0.728(1)	0.25	0.007(5)	7.94(9)
**Li1**	2b	2/3	1/3	0	0.470(2)	0.1
**Mn1**	2b	2/3	1/3	0	0.503(3)	0.1
**Li2**	2b	1/3	2/3	0	0.111(9)	0.1
**Mn2**	2b	1/3	2/3	0	0.845(8)	0.1
**Li3**	2a	0	0	0	0.175(4)	0.1
**Mn3**	2a	0	0	0	0.783(4)	0.1
**O1**	6c	0.047(6)	0.711(6)	0.088(3)	1	0.72(1)
**O2**	6c	0.344(2)	1	0.911(2)	1	0.72(1)

Supplementary Table 3. EDS results of NLMO and NLMO-Zn. The Li content was not considered when calculating the weight and atomic percent.

Sample	Element	Line Type	Wt%	Atomic %	Theoretical Atomic %
**NLMO-Zn**	O	K series	32.26	53.97	55.81
	Na	K series	19.92	23.19	23.26
	Mn	K series	42.07	20.49	19.88
	Zn	L series	5.74	2.35	1.05
**NLMO**	O	K series	32.90	54.03	55.81
	Na	K series	20.89	23.87	23.26
	Mn	K series	46.21	22.10	20.93

Supplementary Table 4. ICP results of the NLMO and NLMO-Zn. The O content was not considered when calculating the atomic percent.

Sample	Element	Weight content (ppm)	Atomic %	Theoretical Atomic %
**NLMO-Zn**	Li	16192	14.70	13.64
	Na	164182	45.48	45.45
	Mn	337652	39.55	38.86
	Zn	45642	0.27	2.05
**NLMO**	Li	17852	15.52	13.64
	Na	171919	45.13	45.45
	Mn	358272	39.35	40.91

Supplementary References

1. Clement, R. J. et al. Structurally stable Mg-doped P2-$Na_{2/3}Mn_{1-y}Mg_yO_2$ sodium-ion battery cathodes with high rate performance: insights from electrochemical, NMR and diffraction studies. *Energy Environ. Sci.* **9**, 3240−3251 (2016).
2. Pang, W.-L. et al. P2-type $Na_{2/3}Mn_{1-x}Al_xO_2$ cathode material for sodium-ion batteries: Al-doped enhanced electrochemical properties and studies on the electrode kinetics. *J. Power Sources* **356**, 80−88 (2017).
3. Billaud, J. et al. β-$NaMnO_2$: A high-performance cathode for sodium-ion batteries. *J. Am. Chem. Soc.* **136**, 17243−17248 (2014).
